# Ethanol Foams Stabilized by Isobutyl-Based POSS–Organosilica Dual-Particle Assemblies

**DOI:** 10.1021/acsami.3c18615

**Published:** 2024-03-04

**Authors:** Kang Wang, Shi Zhang, Dmytro Dedovets, Marc Pera-Titus

**Affiliations:** † Cardiff Catalysis Institute, School of Chemistry, Cardiff University, Main Building, Park Place, Cardiff CF10 3AT, U.K.; ‡ Laboratoire du Futur (LOF), UMR 5258 CNRS-Solvay-Universite Bordeaux 1, 178 Av Dr Albert Schweitzer, 33608 Pessac Cedex, France

**Keywords:** ethanol, foam, organosilica, POSS, dual particle

## Abstract

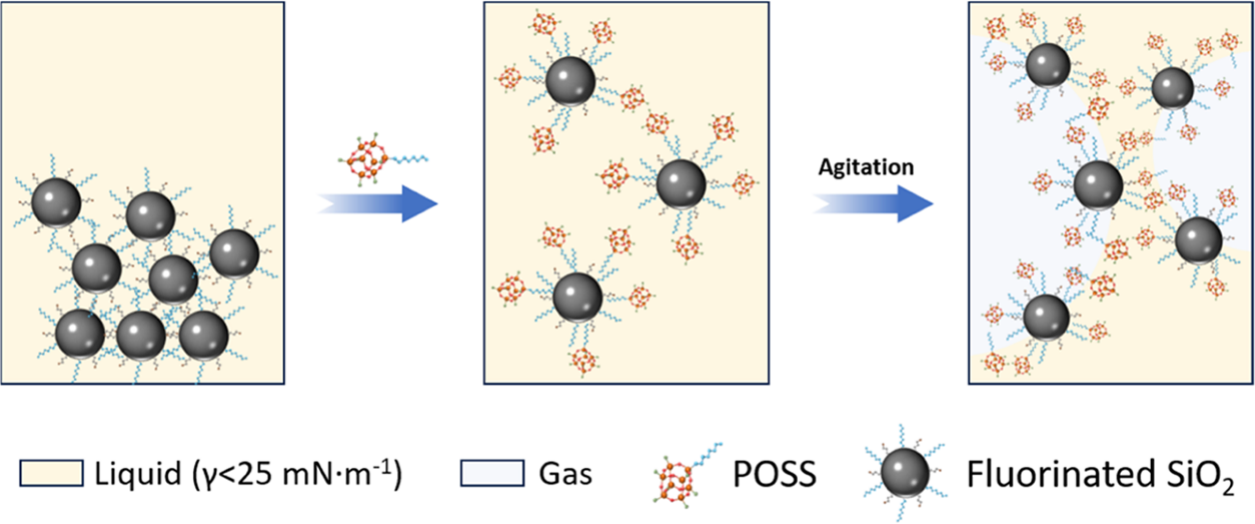

Nonaqueous foams in low-surface tension solvents (<25 mN·m^–1^) are highly desired for applications in fire extinguishers and detoxification gels. However, their formation is a Holy Grail of the chemical industry due to the need for stabilizers with low surface energy and high recyclability. Herein, we disclose a new strategy to generate abundant foams in ethanol and a variety of low-surface tension solvents relying on the interfacial coadsorption of two different particles. The particles consist of surface-active fluorinated silica particles, used as a stabilizer, and a novel amphiphilic polyhedral oligomeric silsesquioxane (POSS) decorated with isobutyl cage substituents, used as a frother. The interaction between POSS and fluorinated particles at the ethanol–air interface was thoroughly investigated by combining physicochemical methods (contact angle, dynamic surface tension, and dynamic light scattering methods) and catalytic tests using the model aerobic oxidation reaction of benzyl alcohol. Both particles could be conveniently recycled for at least 5 consecutive runs with high foamability and catalytic activity.

## 1 Introduction

Nonaqueous foams are a specific type of foams that are produced using a liquid medium other than water as a continuous phase (e.g., hydrocarbons, oils, alcohols) and may contain air or any other gas as a disperse phase. Nonaqueous foams find applications in the cosmetic, oil recovery, and manufacturing industries.^1−3^ Owing to the low surface tension of organic solvents (typically from 14 to 50 mN·m^–1^), the generation of nonaqueous foams requires stabilizers with low surface energy (e.g., fluorinated surfactants, asphaltenes).^4−6^ In particular, the stabilization of foams in solvents with very low surface tension (γ < 25 mN·m^–1^ at 20 °C) (e.g., ethanol, hydrocarbons) has been seldom achieved with foams showing poor stability (<5 min).^7^ Foams in low-surface tension solvents such as ethanol are, however, appealing, given their ability to spread easily and form thin films on surfaces, leading to high wetting and coating properties.

Particle-stabilized aqueous foams have been thoroughly studied during the past decade using a variety of stabilizers such as polymers, proteins, particles, and crystals.^8−11^ However, very few studies have described the preparation of nonaqueous foams owing to their much lower surface tensions, restricting particle adsorption at the gas–liquid (G-L) interface.^12,13^ As a rule, the genesis of particle-stabilized foams relies on the ability of particles to form a jammed or closely packed interfacial armor of particles, preventing the coalescence of gas bubbles and drainage of the liquid phase. To adsorb at the G-L interface and generate foams, successful particles need to meet simultaneously three conditions: (1) the particles need to be overall oleophilic to disperse in the solvent before foaming without agglomeration; (2) the particles need a balanced surface density and distribution of oleophilic and oleophobic (gas-philic) groups to adjust the interfacial contact angle within the “stability window” range, which is much narrower than in particle-stabilized emulsions;^14^ and (3) the particles need a controlled size to diffuse fast from the bulk liquid to the G-L interface.

Examples of nonaqueous foams stabilized by surface-active particles are scarce. Binks and co-workers prepared foams in nonpolar hydrocarbons and polar oils (28–63 mN·m^–1^ at 20 °C) using fluorinated particles such as polytetrafluoroethylene (PTFE) or fluorinated silicas,^13,15−17^ showing stability for several months. Dyab et al. prepared stable foams based on glycerol and ethylene glycol (γ > 47 mN·m^–1^ at 20 °C) using dichlorodimethylsilane-modified silicas.^18^ We also prepared foams in aromatic solvents with intermediate surface tension (35–44 mN·m^–1^ at 20 °C) using biphenyl-bridged organosilica particles decorated with ethoxy (C2) groups that outperformed PTFE and fluorinated surfactants in benzyl alcohol.^19^ Likewise, the combination of fluorinated organosilica particles (oleophobic), used as a stabilizer, and fluorinated anisotropic polyhedral oligomeric silsesquioxane (POSS) with phenyl cage substituents (i.e., Ph_7_/F_13_–POSS) (oleophilic), used as a frother, could stabilize foams in aromatic solvents with a surface tension ranging from 27 to 45 mN·m^–1^ by tuning the wettability of organosilica particles.^20^ Ph_7_/F_13_–POSS nanoparticles (0.1–0.2 wt %) adsorbed on the organosilica particles, embedding the fluorinated chains while exposing phenyl groups into the liquid phase. This promoted the dispersion of organosilica particles in the solvent and allowed fine-tuning of the interfacial contact angle within the “stability window,” resulting in stable foam formation.

Herein, we designed a new dual-particle system combining fluorinated organosilica particles and a novel type of fluorinated POSS decorated with isobutyl cage substituents, allowing for the first time the genesis of stable foams in pure ethanol among other solvents with very low surface tension (γ < 25 mN·m^–1^ at 20 °C). The interaction between organosilica and POSS particles was investigated in detail by combining contact angle, dynamic surface tension, and dynamic light scattering methods and catalytic tests to ascertain the underlying mechanism promoting particle adsorption at the ethanol–air interface.

## 2 Materials and Methods

### 2.1 Materials

Tetraethyl orthosilicate (TEOS, 98%), 1H,1H,2H,2H-perfluorodecyltriethoxysilane (PFDTES, 97%), (3-mercaptopropyl)triethoxysilane (MPTES, >80%), isobutyltriethoxysilane (IBTES, ≥95%), trichloro(1H,1H,2H,2H-perfluorooctyl)silane (97%), 3-Aminopropyltriethoxysilane (APTES, 99%), lithium hydroxide pellets (99%), ammonium hydroxide solution (28–30%), Rhodamine B isothiocyanate (mixed isomer), anhydrous ethanol (99.9%), acetonitrile (99.8%), tetrahydrofuran (99.9%), palladium(II) acetate (98%), and potassium borohydride (KBH_4_, 98%), all purchased from Sigma-Aldrich, were used to prepare the catalytic organosilica particles and isopropyl-based POSS. Benzyl alcohol (>99%), benzaldehyde (99%), anhydrous ethanol (99.9%), acetone (>99.5%), decane (>99%), isopropanol (≥99.5%), dodecane (≥99%), methanol (≥99.8%), dichloromethane (≥99.8%), octane (≥98%), 1-octene (98%), and toluene (≥99.5%), also supplied by Sigma-Aldrich, were used for the foaming tests and oxidation reactions.

### 2.2 Methods

#### 2.2.1 Synthesis of IBu_7_/F_13_–POSS

Isobutyl-based POSS was synthesized by hydrolytic condensation of isobutyltriethoxysilane catalyzed by lithium hydroxide (Figure S1a). Briefly, isobutyltriethoxysilane (23.06 g, 0.105 mol) was added dropwise to lithium hydroxide monohydrate (2.00 g, 0.048 mol) and water (1.60 g, 0.137 mol) in 100 mL of an acetone/methanol mixture (44:6 v/v) at 70 °C under reflux, and the reaction mixture was maintained at this temperature for 18 h. After the reaction, the mixture was cooled to room temperature, and the resulting solid was washed with acetonitrile and then dried at 80 °C overnight. The solid was labeled as IBu_7_-POSS.

IBu_7_/F_13_–POSS was synthesized by a reaction between IBu_7_-POSS and trichloro(1H,1H,2H,2H-perfluorooctyl)silane, allowing corner-capping of Si-OLi groups (Figure S1b). In a typical synthesis, 1.1 g of IBu_7_-POSS was charged into a vial (20 mL) equipped with a magnetic stirrer, and 10 mL of anhydrous tetrahydrofuran was added under vigorous stirring. The vial was then immersed into an ice–water bath, and trichloro(1H,1H,2H,2H-perfluorooctyl)silane (0.368 mL, 0.99 mmol) was injected immediately. The mixture was maintained at 0 °C for 4 h and then increased to room temperature for 20 h. After the synthesis, the reaction mixture was centrifuged to remove the resulting lithium chloride salt, and the supernatant was transferred to a watch glass and heated at 80 °C on a hot plate to evaporate the solvent and other volatiles. The product that remained in the watch glass was washed with anhydrous ethanol by centrifugation 3 times to remove the unreacted silane and IBu_7_-POSS. The obtained white gel was further dried at 80 °C in an oven for 12 h. The resulting solid was labeled as IBu_7_/F_13_–POSS.

#### 2.2.2 Preparation of Pd@SiNP_F_17_(1–3) Particles

Fluorinated organosilica particles (33 wt %F, 364 nm particle size) were synthesized by the Stöber method according to a previous protocol using 1H,1H,2H,2H-perfluorodecyltriethoxy-silane (PFDTES), (3-mercaptopropyl)triethoxysilane (MPTES), and tetraethyl orthosilicate (TEOS) precursors, with TEOS/MPTES and TEOS/PFDTES molar ratios of 16 and 3, respectively.^21^ In a typical synthesis, 2 mL of TEOS, 5.6 mL of deionized water, and 3.2 mL of ammonia were dissolved in 40 mL of ethanol at 40 °C for 5 min. Then, 0.135 mL of MPTES and 1.33 mL of PFDTES were added to the solution. The molar ratio of TEOS/PFDTES was 3:1, with a TEOS/MPTES molar ratio of 16. The reaction was carried out at 40 °C for a further 30 min, and the mixture was subsequently centrifuged to collect the modified particles, which were washed with ethanol 3 times. The collected solid was dried at 80 °C for 10 h. The particles were labeled as SiNP_F_17_(1–3).

Catalytic Pd nanoparticles were further loaded on SiNP_F_17_(1–3) (300 mg) by wet impregnation using an ethanol solution of Pd(OAc)_2_ (1 g·L^–1^). The particle dispersion was stirred mildly at room temperature for 2 h. Then, the particles were isolated by centrifugation and were reduced with KBH_4_ dissolved in 20 mL of ethanol. After mild stirring for 3 h at room temperature, the solid was isolated by centrifugation, washed four times with anhydrous ethanol, and dried at 80 °C for 10 h. The particles were labeled as Pd@SiNP_F_17_(1–3) (1.35 wt % Pd).

#### 2.2.3 Preparation of SiNP_F_17_(1–3)_RB Particles

In the preparation of SiNP_F_17_(1–3)_RB particles, 2 mL of TEOS, 5.6 mL of deionized water, and 3.2 mL of ammonia were dissolved in 40 mL of ethanol at 40 °C for 5 min. Then, 0.133 mL of APTES and 1.33 mL of PFDTES were added to the solution. The molar ratio of TEOS/PFDTES was 3:1, with a TEOS/APTES molar ratio of 16. The reaction was carried out at 40 °C for an additional 30 min, and the mixture was subsequently centrifuged to collect the modified particles, which were washed with ethanol 3 times. The collected solid was dried at 80 °C for 10 h. In a further step, 0.3 g of the above particles were dispersed in 20 mL of ethanol, 10 mg of Rhodamine B isothiocyanate was added into the suspension, and the suspension was stirred at 60 °C for 24 h.^22^ After the reaction, the mixture was washed 3 times with ethanol, the solid was separated by centrifugation, and the precipitate was dried at 80 °C for 10 h. The particles were labeled as SiNP_F_17_(1–3)_RB.

### 2.3 Catalyst characterization

#### 2.3.1 TG Analysis

The thermal profiles of the different particles were measured by thermogravimetric analysis (TGA). Before the tests, the samples (∼10 mg in a 100 μL alumina crucible) were treated from 30 to 900 °C with a heating rate of 10 °C/min under an airflow of 30 mL (STP)/min.

#### 2.3.2 ICP-MS

The Pd composition of the Pd@SiNP_F_17_(1–3) particles was analyzed by inductively coupled plasma (ICP) on an ICP-MS apparatus (PerkinElmer Optima). In a given test, 20 mg of the sample was weighed and loaded in an ICP tube with 4 mL of an aqueous solution of H_2_SO_4_, and the mixture was heated to 150 °C for 20 min. Then, HNO_3_ (10 mL) was added, and the tube was heated to 180 °C for 60 min. After this period, additional HNO_3_ (10 mL) was added, and the tube was kept at 180 °C for 60 min.

#### 2.3.3 ICP-MS

The high-resolution mass spectrometry spectra of IBu_7_/F_13_–POSS were measured on a Xevo G2-XS QTof mass spectrometer using methanol as a solvent.

#### 2.3.4 FT-IR

The Fourier transform infrared spectroscopy (FT-IR) spectra of IBu_7_-POSS and IBu_7_/F_13_–POSS particles were measured from 500 to 4000 cm^–1^ on a Bruker Vertex FT-IR spectrometer with 4 cm^–1^ resolution. Each spectrum was measured after 256 scans.

#### 2.3.5 ^13^C and ^29^Si NMR MAS

Solid-state ^13^C and ^29^Si NMR MAS spectra were acquired on a Bruker AVANCE III 500 spectrometer equipped with a wide bore 11.7 T magnet by using an operational frequency of 500 MHz. A 4 mm triple resonance probe in double resonance mode with magic angle spinning (MAS) was employed in all of the experiments, and the samples were packed on a zirconia rotor and spun at the MAS rate of 15 kHz. The frequency for the ^13^C and ^29^Si NMR MAS was 11 kHz. In the case of ^29^Si NMR MAS, cross-polarization (CP) for proton decoupling was applied. The relaxation delay, d_1_, between accumulations was 5 and 60 s for ^13^C and ^29^Si, respectively. All chemical shifts were reported using the δ scale and were externally referenced to glycine for ^13^C NMR MAS and TMS for ^29^Si NMR MAS. The samples were packed into an NMR rotor and dehydrated at 573 K under vacuum (1 × 10^–4^ mbar) for 2 h prior to loading into the magnet and recording the spectra.

#### 2.3.6 Contact Angles

Contact angles were measured by the sessile drop method on a Dataphysics tensiometer by depositing a small drop of 4 μL of liquid on pellets formed from powder (repeated three times per sample). The pellets were prepared using at least 240 mg of particle powder made by compression under a load of 10 kN using a digital hydraulic press (Pike Technologies) for at least 30 min. The shape of the drops was observed and was used to determine the contact angles.

#### 2.3.7 DLS

Dynamic light scattering (DLS) measurements of particle dispersions were measured using a Malvern Zetasizer Nano ZS particle size analyzer at 25 °C. Around 3 mL of well-dispersed particle dispersion was transferred into a glass cuvette. Each measurement was passed in 8 runs, with each run lasting 30 s. The repeatability for each sample was confirmed after 6 measurements.

#### 2.3.8 Dynamic Surface Tension

Dynamic surface tension of benzyl alcohol and ethanol with various particle concentrations was measured using a bubble pressure tensiometer-BP100 (KRÜSS). Typically, calibration was carried out with ultrapure water at 25 °C to obtain a precise capillary diameter. Before each measurement, 50 mL of a particle solution was prepared and dispersed well by sonication. Then, the solution was transferred into an SV20 glass vessel (121.5 mL, 70 mm) with a 66.5 mm diameter top, 66.5 mm diameter bottom, and 35 mm height center. The surface tension measurements were conducted using a 20 mm/min detection speed, 50 Pa detection sensitivity, and an initial surface age of 10 ms.

### 2.4 Foaming Tests

The foaming tests were carried out in an 8 mL glass vial (o.d. 17 mm, height 60 mm) with 1 mL of liquid. Different foaming methods were tested, including handshaking, ultra-turrax (IKA T-10, 30 000 rpm, 30 s), ultrasonic probe (Sonics VCX750, 20% amplitude, pulse 15/15 s), and magnetic stirring (3 mm × 13 mm stirring bar) at 1500 rpm for 30 min. In these methods, variable amounts of IBu_7_/F_13_–POSS and SiNP_F_17_(1–3) particles were added to the vial. The foamability (i.e., foam height, measured with a ruler) and foam stability were measured immediately after aeration and monitored statically as a function of time. The bubble size distribution was measured using a Leica DM750 optical microscope with GXCAM software, 10× ocular, 4 ×, 10 ×, 40 x, and 100× objectives. ImageJ software was used to quantify the average droplet size.

### 2.5 Catalytic Tests

The oxidation of benzyl alcohol in ethanol was conducted in a batch reactor under O_2_ using a balloon (1 L) at ambient pressure. In a typical test, BnOH (50 mg, 0.46 mmol), ethanol (1 g), Pd@SiNP_F_17_(1–3) (40 mg), and a given amount of IBu_7_/F_13_–POSS (0–1 mg) were added to a Schlenk tube (25 mL) with a magnetic stirring bar. The Schlenk tube was purged three times with O_2_, and the inlet tube was connected to the O_2_ balloon. Then, the Schlenk tube was connected to a condenser with a silicon rubber cap on top. In a typical test, the oxidation reaction was carried out at 60 °C for 60 min with stirring (1500 rpm). After the reaction, acetone was added to destabilize the as-generated foam, the liquid was centrifuged (7000 rpm) for 3 min, and the supernatant solution was recovered using a syringe to run the GC analyses.

The solution, after the reaction, was analyzed using an Agilent 7820A GC equipped with a flame ionization detector (FID) and an HP-5 column (length 30 m, i.d. 0.25 mm). Mass balance errors were within 5% for all of the catalytic tests. The BnOH conversion and benzaldehyde (BnAH) yield were calculated by interpolation of the corresponding calibration curves using biphenyl as the internal standard as follows
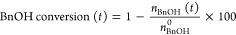
1
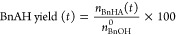
2where *n*_BnOH_^0^ and *n*_BnOH(t)_ refer to the mole number of BnOH at time = 0 and time = *t*, respectively, and *n*_BnAH_(*t*) is the mole number of BnAH at time = *t*.

### 2.6 Catalyst Reusability Tests

A series of reusability tests were performed to evaluate the stability of Pd@SiNP_F_17_(1–3) and IBu_7_/F_13_–POSS particles in 5 consecutive catalytic runs. The tests were carried out at 60 °C for 1 h under stirring (1500 rpm) using 50 mg of BnOH, 4 wt % Pd@SiNP_F_17_(1–3), and 0.2 wt % IBu_7_/F_13_–POSS (in 1 g of ethanol). After each catalytic run, 2 mL of deionized water was added to the reaction solution, and the Pd@SiNP_F_17_(1–3) and IBu_7_/F_13_–POSS particles were separated together from the reaction media by centrifugation (7000 rpm for 3 min), washed three times with water, and dried at 80 °C in a vacuum oven for at least 4 h before reuse in the subsequent run.

## 3 Results and Discussion

### 3.1 Preparation of IBu_7_/F_13_–POSS Nanoparticles

**Figure 1:**
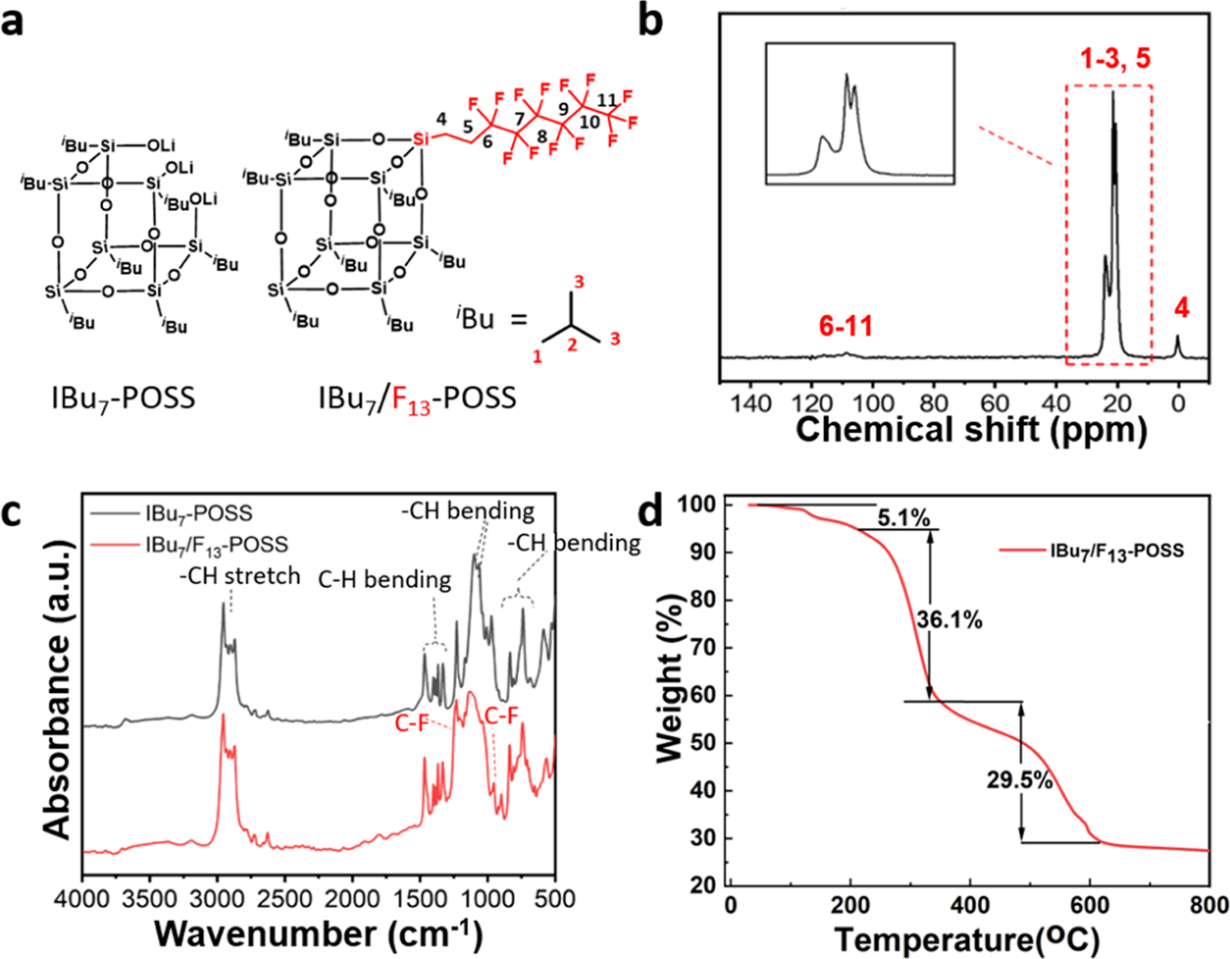
(a) Molecular structure of IBu_7_-POSS and IBu_7_/F_13_–POSS. (b) ^13^C NMR MAS spectra of IBu_7_/F_13_–POSS. (c) FT-IR spectra of IBu_7_-POSS and IBu_7_/F_13_–POSS. (d) TGA of IBu_7_/F_13_–POSS.

Isobutyl-based POSS (i.e., IBu_7_-POSS) was synthesized by hydrolytic condensation of isobutyltriethoxysilane catalyzed by lithium hydroxide (see the ESI for experimental details). The sample was further functionalized with perfluorocarbon chains by reaction with trichloro(1H,1H,2H,2H-perfluorooctyl) silane, allowing corner-capping of Si-OLi groups (i.e., IBu_7_/F_13_–POSS). The molecular structure of IBu_7_-POSS and IBu_7_/F_13_–POSS (Figure 1a) was confirmed by MS showing the presence of a characteristic molecular ion peak at *m*/*z* = 1163.26 matching the theoretical value (*m*/*z* = 1163.27) (Figure S2).

The solid-state ^13^C NMR MAS spectrum of IBu_7_/F_13_–POSS (Figure 1b) displays a resonance band at 2.1 ppm that can be assigned to the carbon close to silicon in the perfluorocarbon chain.^23^ Additional bands are visible between 17.7 and 26.8 ppm that correspond to CH_2_ groups in the isobutyl and perfluorocarbon chains.^24^ Besides, a broad and complex band appears in the range 110–120 ppm that can be assigned to CF_3_ and CF_2_ groups in the perfluorocarbon chains that confirm their successful grafting on capped Si-OLi groups.^23^ The solid ^29^Si NMR MAS (Figure S3) exhibits the presence of a single band at a chemical shift of −67.4 ppm falling into the T^3^ region that confirms the closure of IBu_7_/F_13_–POSS cages in the corner-capping reaction.^25^

The liquid ^19^F NMR spectrum of IBu_7_/F_13_–POSS (Figure S4) displays a characteristic band at 80.7 ppm that is ascribed to CF_3_ groups in the perfluorocarbon chain.^26^ Additional sharp bands are visible in the range 115–128 ppm that can be attributed to internal CF_2_ groups in the chains.^26^ The liquid ^1^H NMR spectrum of IBu_7_/F_13_–POSS (Figure S5) exhibits characteristic bands at 0.6, 0.9, and 1.8 ppm that are attributed to CH_2_ groups in the isobutyl groups, whereas additional bands centered at 0.7 and 2.1 ppm are indicative of CH_2_ groups in the perfluorocarbon chain.

IBu_7_-POSS and IBu_7_/F_13_–POSS were also analyzed by FT-IR spectroscopy (Figure 1c). The spectra show characteristic bands at 800 and 1100 cm^–1^ that are attributed to the asymmetric stretching and bending vibrations of Si–O–Si bonds. Characteristic bands of IBu_7_/F_13_–POSS due to the fluorocarbon chain appear at 913, 1171, and 1237 cm^–1^ that can be assigned to stretching modes of C–Si and C–F (CF_2_/CF_3_) bonds, respectively.^27^ Additional bands at 710 and 670 cm^–1^ are attributed to symmetric stretching bands of CF_3_ groups.^28^ The bands that are visible between 2750 and 3000 cm^–1^ can be attributed to C–H stretching vibrations of isobutyl groups.^29^

The TG profile of IBu_7_/F_13_–POSS exhibits a small weight loss (about 5.1%) below 210 °C that is attributed to water desorption (Figure 1d). The weight loss between 210 and 350 °C (about 36.1%) is compatible with the presence of 7 pendant isobutyl groups on the POSS cage (theoretical weight loss = 34.3%). The sample also exhibits a weight loss between 350 and 610 °C (about 29.5%) that can be ascribed to the combustion of the perfluorocarbon chain (theoretical weight loss = 29.8%). This observation further demonstrates the grafting of the perfluorocarbon chain on IBu_7_-POSS.^30^

### 3.2 Foaming Tests over Combined IBu_7_/F_13_–POSS and Fluorinated Organosilica Particles

**Figure 2:**
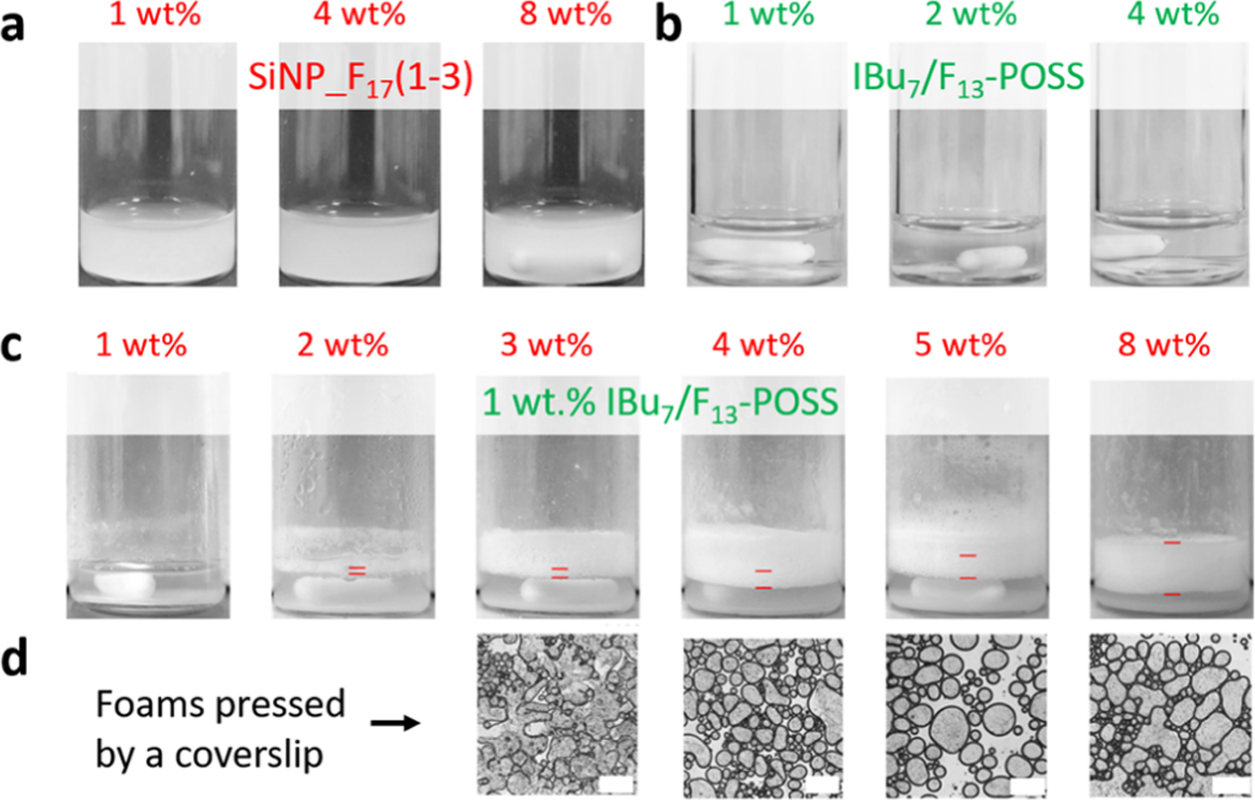
Foaming tests in ethanol using (a) 1–8 wt % SiNP_F_17_(1–3), (b) 1–4 wt % IBu_7_/F_13_–POSS, and (c) 1–8 wt % of SiNP_F_17_(1–3) at constant IBu_7_/F_13_–POSS concentration (1 wt %). (d) Microscope images of the foams pressed by the coverslip. Foaming conditions: 25 °C, stirring at 1500 rpm for 30 min, stabilization for 1 min before visualization. The scale bar in (d) is 1000 μm.

The foaming properties of IBu_7_/F_13_–POSS in anhydrous ethanol (γ = 22 mN·m^–1^ at 20 °C) were inspected at room temperature either alone or combined with oleophobic organosilicas [SiNP_F_17_(1–3)] (33 wt % F, 364 nm particle size, see TG profile in Figure S6). The foams were prepared using four different methods (Figure S7, see the ESI for details): (1) handshaking, (2) ultra-turrax, (3) ultrasonic probe, and (4) magnetic stirring (1500 rpm for 30 min). Magnetic stirring provided the best foamability, and accordingly, this method was used hereinafter to measure the foaming properties of the particles. IBu_7_/F_13_–POSS nanoparticles alone (1–4 wt %) can disperse in ethanol and generate foams by either handshaking or stirring but vanish almost instantly, even at 4 wt % concentration (Figure 2b). This observation points to a lack of IBu_7_/F_13_–POSS adsorption at the ethanol–air interface. We also explored the foaming properties of SiNP_F_17_(1–3) particles after stirring at 1500 rpm. The particles can be wetted and dispersed in ethanol but display no foamability in the range of 1–8 wt % (Figure 2a).

Inspired by the already observed synergy between Ph_7_/F_13_–POSS and Pd@SiNP_F_17_ particles for generating foams in aromatic alcohols (γ > 30 mN·m^–1^ at 20 °C),^10^ we combined IBu_7_/F_13_–POSS and SiNP_F_17_(1–3) particles to assess their ability for stabilizing foams in ethanol. Keeping the IBu_7_/F_13_–POSS concentration at 1 wt %, adding SiNP_F_17_(1–3) particles at a concentration below 3 wt % generates a tiny foam layer that vanishes within 1 h (Figures 2c and S8). In contrast, thicker ethanol foams are generated above 3 wt % SiNP_F_17_(1–3) that keep stable for at least 2 h with a foamability (i.e., foam height) increasing with the SiNP_F_17_(1–3) concentration. When visualized by optical microscopy, gas bubbles are not resistant against compression by the coverslip below 3 wt % SiNP_F_17_(1–3), suggesting poor interfacial coverage by SiNP_F_17_(1–3) and IBu_7_/F_13_–POSS assemblies (Figure 2d). In contrast, above 4 wt % SiNP_F_17_(1–3), the bubbles are highly resistant, and further increase of the SiNP_F_17_(1–3) concentration to 5–8 wt % generates dense and robust ethanol foams with an average bubble size of in all cases about 200 μm. A representative bubble size distribution is provided in the SI (Figure S9).

**Figure 3:**
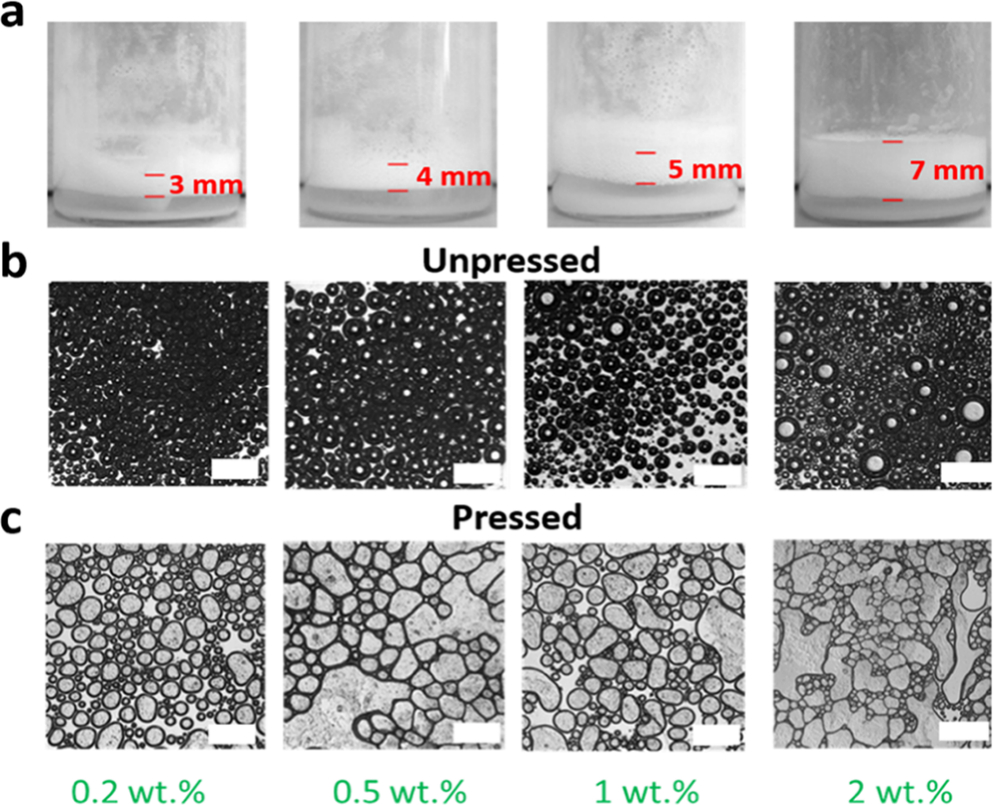
(a) Optical images of foams in ethanol using 4 wt % SiNP_F_17_(1–3) at variable IBu_7_/F_13_–POSS concentration (0.2–2 wt %). (b) Microscopic images of foams in ethanol using 4 wt % SiNP_F_17_(1–3) at variable IBu_7_/F_13_–POSS concentration (0.2–2 wt %). (c) Microscopic images of foams in ethanol using 4 wt % SiNP_F_17_(1–3) at variable IBu_7_/F_13_–POSS concentration (0.2–2 wt %) pressed by a coverslip. Foaming conditions: 25 °C, stirring for 30 min at 1500 rpm, and stabilization for 5 min before visualization. The scale bar in the optical images is 1000 μm.

We next investigated the influence of the IBu_7_/F_13_–POSS concentration (range 0.2–4 wt %) at constant SiNP_F_17_(1–3) concentration (4 wt %) on the ethanol foaming properties (Figures 3 and S10). The foamability increases monotonically with the IBu_7_/F_13_–POSS concentration from 3 to 7 mm (Figure 3a). Further monitoring of the time-evolution of the foam height using 5 wt % SiNP_F_17_(1–3) and 0.2–2 wt % IBu_7_/F_13_–POSS reveals high stability for 72 h with only moderate decline of the foam height (Figure S11a,b). Inspection of the time-evolution of normalized heights reveals slightly higher stability at higher IBu_7_/F_13_–POSS concentrations (Figure S11c). In line with these observations, the average size of bubbles remains almost unchanged while increasing the IBu_7_/F_13_–POSS concentration (Figure 3b). However, the bubbles become more fragile after being pressed by the coverslip (Figure 3c). This observation can be explained by a lower loading of adsorbed SiNP_F_17_(1–3) particles at the ethanol–air interface at a higher IBu_7_/F_13_–POSS concentration due to higher interfacial competition between both particles.

To appraise the relative adsorption between IBu_7_/F_13_–POSS and SiNP_F_17_(1–3) particles at the ethanol–air interface, we synthesized fluorescent fluorinated organosilica particles decorated with rhodamine b isothiocyanate groups (i.e., SiNP_F_17_(1–3)_RB, see the ESI for details). We then prepared foams by combining 0.5 wt % IBu_7_/F_13_–POSS and 8 wt % SiNP_F_17_(1–3)_RB after stirring at 1500 rpm for 30 min and visualized the bubbles by optical microscopy (Figure S12). The presence of fluorescent particles is clearly observed at the boundary of bubbles with high densification. This observation confirms the adsorption of SiNP_F_17_(1–3) particles at the ethanol–air interface in the presence of IBu_7_/F_13_–POSS nanoparticles.

### 3.3 Understanding the Interaction between IBu_7_/F_13_–POSS and SiNP_F_17_(1–3) Particles

To rationalize the interaction between IBu_7_/F_13_–POSS and SiNP_F_17_(1–3) particles at the ethanol–air interface and the mechanism promoting the genesis of ethanol foams, we combined contact angle, dynamic surface tension, and dynamic light scattering measurements, together with catalytic tests.

#### 3.3.1 Interfacial Contact Angle and Dynamic Surface Tension

We measured the interfacial contact angles and dynamic surface tension of ethanol in the presence of single and combined IBu_7_/F_13_–POSS and SiNP_F_17_(1–3) particles. SiNP_F_17_(1–3) particles alone show a contact angle of 40° with ethanol, whereas the contact angle for IBu_7_/F_13_–POSS nanoparticles is 53° (Figure S13). This observation points out a lower dispersion of IBu_7_/F_13_–POSS nanoparticles when suspended in ethanol.

Dynamic surface tension tests in ethanol and BnOH exhibit, in both cases, a decrease of the surface tension, pointing out the surface-active (i.e., surfactant-like) properties of IBu_7_/F_13_–POSS (Figure S14). The decline of surface tension is higher for BnOH evolving from 40 mN·m^–1^ without IBu_7_/F_13_–POSS to 27.5 mN·m^–1^ at 20 °C using 0.5 wt % IBu_7_/F_13_–POSS (Figure S14a). In the case of ethanol, despite its much lower surface tension (22.3 mN·m^–1^ at 20 °C), a slight reduction to 21.9 mN·m^–1^ (20 °C) is observed (Figure S14b). In light of these results, the surface-active nature of IBu_7_/F_13_–POSS nanoparticles is expected to promote the diffusion of SiNP_F_17_(1–3) particles to the ethanol–air interface during the foaming process. More details are provided in the subsections below.

#### 3.3.2 Dynamic Light Scattering

The dispersion of the particles in ethanol was further studied by dynamic light scattering (DLS). When dispersed in ethanol, SiNP_F_17_(1–3) (4 wt %) particles show an average particle size of ∼1000 nm that reveals partial agglomeration when compared to the nominal particle size (364 nm) (Figure S15a). Larger agglomeration is observed for IBu_7_/F_13_–POSS nanoparticles (0.2 wt %) with an average particle size of 170 nm. Using THF as a solvent, IBu_7_/F_13_–POSS nanoparticles exhibit higher dispersion with an average particle size below 10 nm at low IBu_7_/F_13_–POSS concentration (0.001 wt %), whereas small aggregates (34 nm) are observed at higher particle concentration (0.1 wt %) (Figure S15c). These observations point out that ethanol is a bad dispersant of both particles when being suspended alone.

Opposing the observations above, when IBu_7_/F_13_–POSS and SiNP_F_17_(1–3) particles are dispersed together in ethanol, the average particle size is ∼410 nm, which is only slightly higher than the sizes measured for SiNP_F_17_(1–3) and IBu_7_/F_13_–POSS particles alone (Figure S15a), and approaches the nominal size of SiNP_F_17_(1–3) particles (364 nm). This value remains almost unchanged regardless of the IBu_7_/F_13_–POSS concentration (range 0.2 or 2.0 wt %) for 4 wt % SiNP_F_17_(1–3) concentration. Additional DLS measurements at the same particle concentrations but using variable sonication times exhibit a drastic decline of the particle size from 1000 to 410 nm after 10 min of sonication (Figure S15b). These observations confirm the synergistic effect between both particles in ethanol, leading to complete dispersion of SiNP_F_17_(1–3) in the presence of IBu_7_/F_13_–POSS. The slightly higher particle size measured by DLS compared to the nominal SiNP_F_17_(1–3) particle size measured by HR-TEM (410 vs 364 nm) can be explained by partial particle solvation and IBu_7_/F_13_–POSS adsorption on SiNP_F_17_(1–3) particles when dispersed in ethanol.

#### 3.3.3 Catalytic Tests

**Figure 4:**
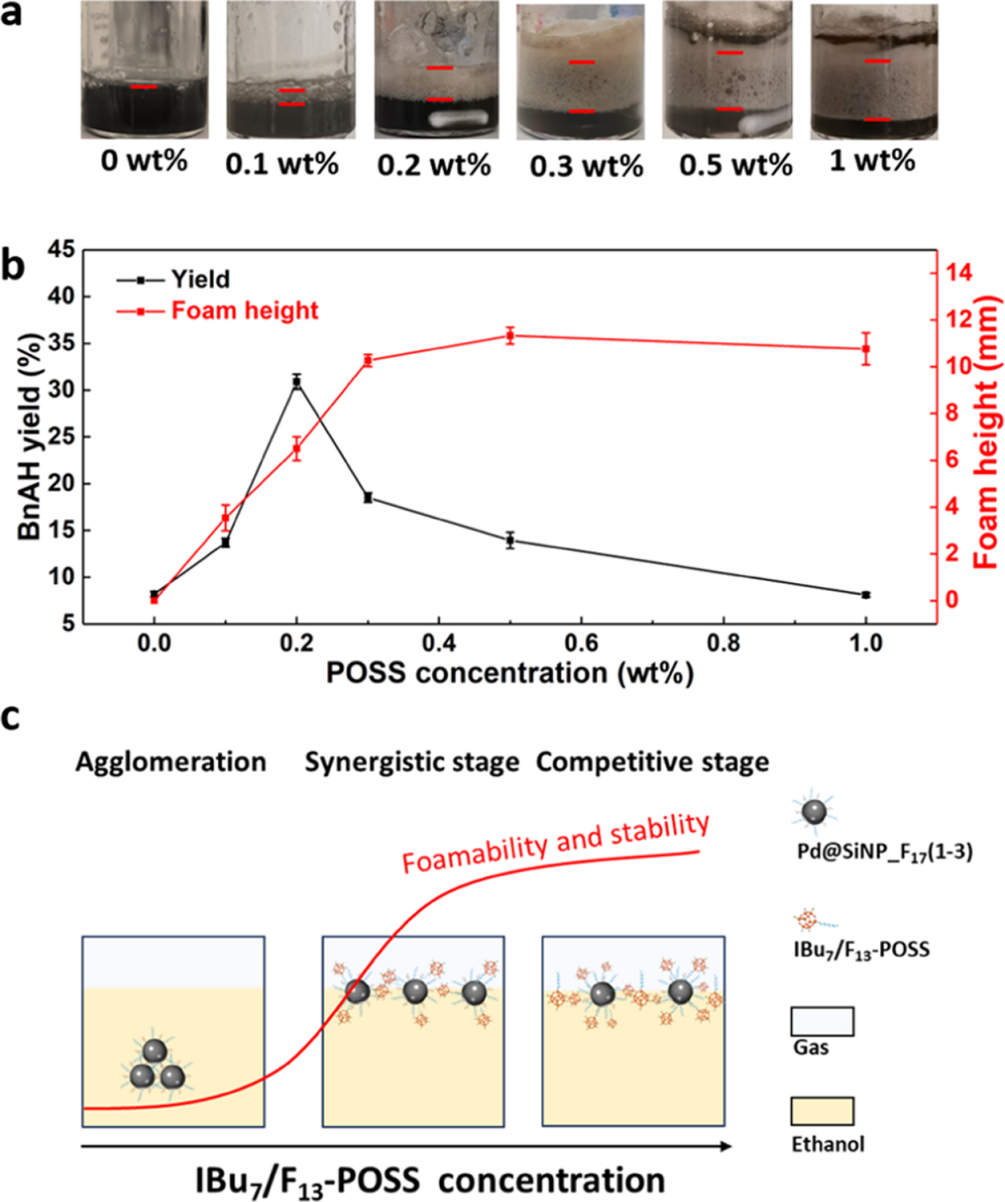
(a) Evolution of the foamability of the Pd@SiNP_F_17_(1–3) and IBu_7_/F_13_–POSS biparticle system in ethanol as a function of the IBu_7_/F_13_–POSS concentration. Foaming conditions: 60 °C, 4 wt % Pd@ SiNP_F_17_(1–3), stirring for 30 min at 1500 rpm, and stabilization for 1 h (reaction time) before visualization. (b) Aerobic oxidation of BnOH over Pd@SiNP_F_17_(1–3) at variable IBu_7_/F_13_–POSS concentration (0–1.0 wt %). Reaction conditions: 1 g of ethanol, 50 mg of BnOH (5 wt %. 0.40 mM), 4 wt % Pd@SiNP_F_17_(1–3), 60 °C, ambient O_2_ pressure (balloon), and stirring at 1500 rpm for 1 h. (c) Schematic representation of the synergy between IBu_7_/F_13_–POSS and Pd@SiNP_F_17_(1–3) particles as a function of the IBu_7_/F_13_–POSS concentration.

We further characterized the SiNP_F_17_(1–3) and IBu_7_/F_13_–POSS dual-particle self-assemblies at the ethanol–air interface using the aerobic oxidation of benzyl alcohol (BnOH) as a model reaction. The reaction was conducted using diluted BnOH in ethanol (5 wt %, 0.40 mM) at 60 °C. To render SiNP_F_17_(1–3) catalytic, Pd nanoparticles were loaded by wet impregnation using an ethanolic solution of Pd(OAc)_2_ (see the ESI for details). The final particles were denoted as Pd@SiNP_F_17_ (1.33 wt % Pd). In the catalytic tests, the Pd@SiNP_F_17_(1–3) concentration was kept constant at 4 wt %, while the effect of the IBu_7_/F_13_–POSS concentration was studied from 0 to 1.0 wt %. Without IBu_7_/F_13_–POSS, the benzaldehyde (BAH) yield is only 8% after 1 h and increases to 32% after adding 0.2 wt % IBu_7_/F_13_–POSS (Figure 4b). Further increase of the IBu_7_/F_13_–POSS concentration to 1 wt % results in a marked decline of the BAH yield down to 8%. Overall, a volcano-type trend is observed for the BAH yield as a function of the IBu_7_/F_13_–POSS concentration with a maximum BAH yield at 0.2 wt %.

To rationalize the trend above, we measured the ethanol foamability and foam stability under the reaction conditions (60 °C, 1 h stabilization) for the Pd@SiNP_F_17_(1–3) and IBu_7_/F_13_–POSS dual-particle system as a function of the IBu_7_/F_13_–POSS concentration. The foam height increases drastically upon addition of IBu_7_/F_13_–POSS from 0 to 1.0 wt %, reaching a plateau of ∼10 mm at 0.3 wt % that keeps constant until 1.0 wt % (Figure 4a). The foam height keeps stable along the reaction (60 °C, 1 h) using 4 wt % Pd@SiNP_F_17_(1–3) and 0.2 wt % IBu_7_/F_13_–POSS and decreases only slightly after 24 h and by reducing the temperature to 25 °C (Figure S16).

Figure 4c presents a schematic representation of the foamability and foam stability for the Pd@SiNP_F_17_(1–3) and IBu_7_/F_13_–POSS dual-particle system as a function of the IBu_7_/F_13_–POSS concentration. Without IBu_7_/F_13_–POSS, Pd@SiNP_F_17_(1–3) particles exhibit agglomeration, as suggested by the DLS plots (Figure S15). By adding a low concentration of IBu_7_/F_13_–POSS (0.2 wt %), Pd@SiNP_F_17_(1–3) can disperse in ethanol driven by the synergy between both particles with no observable entropic depletion, and the particle assemblies can adsorb at the ethanol–air interface after stirring and thus generate stable foams. Further increase of the IBu_7_/F_13_–POSS concentration results in higher foamability and foam stability. In light of this observation, the volcano plot observed for the BAH yield (Figure 4b) can be explained by a lower interfacial density of fluorinated particles when increasing the IBu_7_/F_13_–POSS concentration. This leads to a reduction of available catalytic sites for the reaction, resulting in a lower catalytic activity. This deactivation mechanism differs from that already reported for the Ph_7_/F_13_–POSS + Pd@SiNP_F_17_ dual-particle system, where addition of Ph_7_/F_13_–POSS (>0.1 wt %) results in a pronounced decline of foam stability.^22^

### 3.4 Scope of Low-Surface Tension Solvents

With these results in hand, we examined the generality of combined IBu_7_/F_13_–POSS and SiNP_F_17_(1–3) particles to generate stable foams for a library of solvents with surface tension in the range 22–28 mN·m^–1^ (20 °C), including polar solvents such as methanol, isopropanol, acetone, and dichloromethane, as well as hydrocarbons such as octane, decane, dodecane, 1-octene, toluene, and alcohol mixtures. For comparison, we also tested solvents with higher surface tension (BnOH, ethylene glycol)

**Figure 5:**
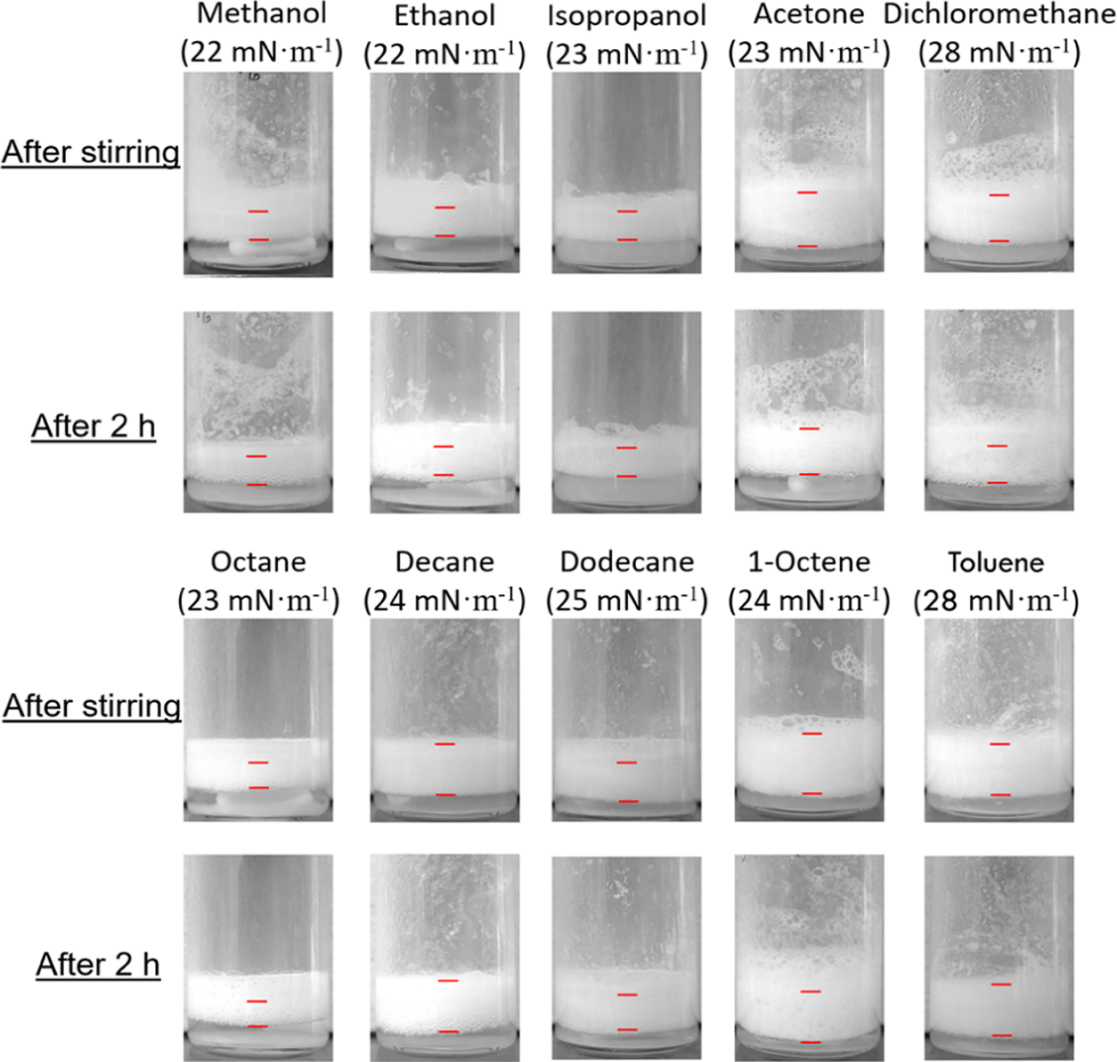
Foaming tests in different low-surface tension solvents (polar and hydrocarbons) using combined 1 wt % IBu_7_/F_13_–POSS and 5 wt %SiNP_F_17_(1–3) particles. Foaming conditions: 25 °C, stirring at 1500 rpm for 30 min, stabilization for 1 min, and 2 h before visualization.

In analogy to ethanol, IBu_7_/F_13_–POSS nanoparticles can disperse in the different solvents and generate foams by either handshaking or stirring. However, their stability is ephemeral. Likewise, SiNP_F_17_(1–3) particles can be wetted and dispersed in the solvents but do not exhibit foamability. By combining 1 wt % IBu_7_/F_13_–POSS and 5 wt % SiNP_F_17_(1–3) particles, foams are generated after stirring at 1500 rpm for 30 min that keep stable for at least 2 h (Figure 5). Likewise, the combination of both particles promotes the generation of foams in alcohol mixtures for aromatic and long-chain aliphatic alcohols (e.g., 1-hexanol, 1-octanol, 1-dodecanol, 1-hexadecanol, 2-octanol) in ethanol (5 wt %) (Figure S17). The dual-particle system can also generate foams in pure BnOH with a surface tension of 39 mN/m (25 °C). However, no foams are produced in very polar ethylene glycol with a surface tension of 48 mN/m (25 °C) due to the poor dispersion of SiNP_F17(1–3) and IBu7/F13-POSS (Figure S18).

Overall, these observations point out the versatility of the IBu_7_/F_13_–POSS and SiNP_F_17_(1–3) dual-particle system for stabilizing foams in solvents with low surface tension and combinations of alcohols with low surface tension. As in the case of ethanol, IBu_7_/F_13_–POSS promotes the dispersion of SiNP_F_17_(1–3) particles in the different solvents as inferred from DLS measurements (Figure S19). The average particle size approaches that of the nominal SiNP_F_17_(1–3) particles (range of 383–499 vs 364 nm) (Table S1).

### 3.5 Particle Recycling

We studied the recyclability of IBu_7_/F_13_–POSS and Pd@SiNP_F_17_(1–3) particles for the aerobic oxidation of BnOH in ethanol foam for five consecutive runs (see the ESI for details). Two different particle separation methods were implemented (see scheme in Figure S20).^20^ First, the IBu_7_/F_13_–POSS and Pd@SiNP_F_17_(1–3) dual-particle system mixture was separated after each run by adding twice the volume of deionized water. This protocol promotes IBu_7_/F_13_–POSS precipitation from the ethanol–water mixture and further separation by centrifugation (Figure S20a). The dual-particle mixture was then dried at 80 °C for 4 h and was used for a subsequent catalytic run.

**Figure 6:**
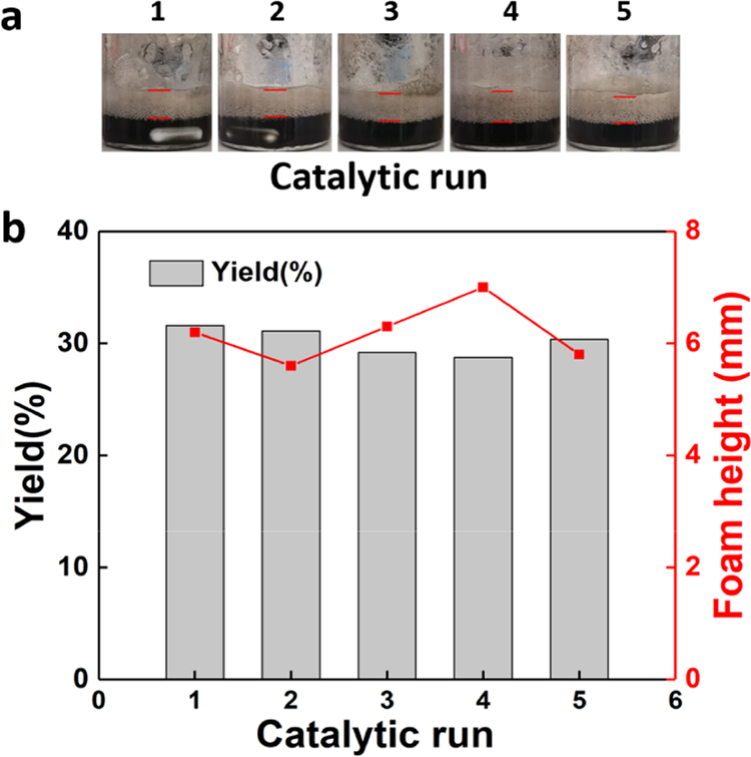
(a) Optical images of ethanol foams after each catalytic run. (b) Recyclability and reuse of Pd@SiNP_F_17_(1–3) and IBu_7_/F_13_–POSS dual-particle systems for the aerobic oxidation of BnOH over five consecutive runs. Reaction conditions: 1 g of ethanol, 50 mg of BnOH, 4 wt % Pd@SiNP_F_17_(1–3), 0.2% IBu_7_/F_13_–POSS, 60 °C, O_2_ balloon, and stirring at 1500 rpm for 1 h.

Besides, to recycle both particles separately, Pd@SiNP_F_17_(1–3) was separated by centrifugation, while IBu_7_/F_13_–POSS remained in the supernatant (Figure S20b). The IBu_7_/F_13_–POSS was then precipitated by adding deionized water, and the nanoparticles were separated by centrifugation. The IBu_7_/F_13_–POSS nanoparticles were further used in the subsequent catalytic run after remixing with Pd@SiNP_F_17_(1–3). In both cases, the Pd@SiNP_F_17_(1–3) and IBu_7_/F_13_–POSS dual-particle system can be reused for at least five consecutive runs without appreciable loss of catalytic activity and foamability (Figure 6). No Pd leaching is observed after the reaction, as inferred by ICP-MS.

## 4 Conclusions

In summary, we prepared foams in a series of low-surface tension solvents stabilized by a dual-particle system consisting of surface-active oleophobic silica particles, used as a stabilizer, and a novel type of amphiphilic polyhedral oligomeric silsesquioxane (IBu_7_/F_13_–POSS) nanoparticle decorated with isobutyl cage substituents, used as a frother. The foamability in low-surface tension solvents was drastically promoted in the presence of a low IBu_7_/F_13_–POSS concentration (0.2–4 wt %) compared to a suspension with oleophobic organosilica particles alone. The addition of IBu_7_/F_13_–POSS helped organosilica particles to disperse in ethanol and promoted interfacial self-assembly after stirring, resulting in stable and abundant foam formation. Further increase of the IBu_7_/F_13_–POSS concentration generated thicker and more stable foams that hindered the particle film permeability and, in turn, the catalytic activity. Particles were conveniently recycled with comparable foamability and foam stability, and the catalytic activity was maintained for at least five consecutive runs. The results presented in this study pave the way to the design of on-purpose, adjustable dual-particle systems for generating nonaqueous foams *à la carte* for a large variety of solvents with low surface tensions.

## Supporting Information Available

Synthesis of IBu_7_-POSS and IBu_7_/F_13_–POSS; mass spectrogram, ^29^Si NMR MAS spectra, liquid ^19^F NMR/^1^H NMR spectra, and TGA profiles of IBu_7_/F_13_–POSS; ethanol foams prepared with SiNP_F_17_(1–3) and IBu_7_/F_13_–POSS, both alone and combined; optical images of ethanol foams with/without coverslip at different magnifications; representative bubble size distribution of foams stabilized by SiNP_F_17_(1–3) and IBu_7_/F_13_–POSS particles; time-evolution of ethanol foams stabilized by SiNP_F_17_(1–3) and IBu_7_/F_13_–POSS particles; optical images of ethanol foams stabilized by IBu_7_/F_13_–POSS + SiNP_F_17_(1–3)_RB particles; contact angle of SiNP_F_17_(1–3), IBu_7_/F_13_–POSS, and combined SiNP_F_17_(1–3) + IBu_7_/F_13_–POSS; dynamic surface tension tests of ethanol and benzyl alcohol in the presence of IBu_7_/F_13_–POSS; DLS tests of IBu_7_/F_13_–POSS and SiNP_F_17_(1–3) particles (single and mixtures) dispersed in ethanol and THF; time-evolution of ethanol foamability by IBu_7_/F_13_–POSS + Pd@SiNP_F_17_(1–3) particles during and after the aerobic oxidation reaction of BnOH; foaming tests for benzyl alcohol and combinations of long-chain aliphatic alcohols dissolved in ethanol using combined 1 wt % IBu_7_/F_13_–POSS and 5 wt %SiNP_F_17_(1–3) particles; DLS tests of single and combined IBu_7_/F_13_–POSS and SiNP_F_17_(1–3) particles in different solvents; and schematic representation of particle recycling (PDF)
