# Supplementary material for: Ethanol Foams Stabilized by Isobutyl-Based POSS–Organosilica Dual-Particle Assemblies
Source: ACS Appl Mater Interfaces. 2024 Mar 4;16(10):13282–90. doi: 10.1021/acsami.3c18615 (PMC10941061; doi:10.1021/acsami.3c18615)
Supplement: Supplementary file 1 — am3c18615_si_001.pdf [file am3c18615_si_001.pdf]

Supporting Information

Ethanol foams stabilized by isobutyl-based POSS-organosilica dual-particle assemblies

Kang Wang,[1] Shi Zhang,[2] Dmytro Dedovets[2] and Marc Pera-Titus[1]*

[1] Cardiff Catalysis Institute, School of Chemistry, Cardiff University, Main Building, Park Place, Cardiff CF10 3AT, UK

[2] Laboratoire du Futur (LOF), UMR 5258 CNRS-Solvay-Universite Bordeaux 1,178 Av Dr Albert Schweitzer, 33608 Pessac Cedex, France

* Corresponding author: peratitusm@cardiff.ac.uk

Table of Contents

TABLE CAPTIONS:

Table S1. Average particle sizes measured by DLS for IBu_7/F_{13}-POSS, SiNP_F_{17}(1-3), and 0.2 wt% IBu_7/F_{13}-POSS and 4 wt% SiNP_F_{17}(1-3) particle dispersions in different solvents.

FIGURE CAPTIONS:

Figure S1. (a) Synthesis of IBu_7-POSS. **(b)** Synthesis of IBu_7/F_{13}-POSS.

Figure S2. Mass spectrogram of IBu_7/F_{13}-POSS.

Figure S3. ^{29}Si NMR MAS spectra of IBu_7/F_{13}-POSS.

Figure S4. Liquid ^{19}F NMR spectrum of IBu_7/F_{13}-POSS.

Figure S5. Liquid ^{1}H NMR spectrum of IBu_7/F_{13}-POSS. $CDCl_3$ was used as solvent.

Figure S6. TGA profiles of SiNP_F_{17}(1-3) particles.

Figure S7. Ethanol foams prepared by (a) handshaking, (b) ultra-turrax, (c) ultrasonication, and (d) after magnetic stirring at 1500 rpm for 30 min. Foaming conditions: 5 wt% SiNP_F_{17}(1-3), 1% IBu_7/F_{13}-POSS, 1 g ethanol, 25 °C, stabilization for 5 min before visualization.

Figure S8. Optical images of ethanol foams with/without coverslip at different magnifications (the unit of scale bar is μm) after stirring. Foaming conditions: 1wt% IBu_7/F_{13}-POSS, 1-8wt% SiNP_F_{17}(1-3), 1 g ethanol, 25 °C, stirring at 1500 rpm for 30 min, stabilization for 5 min before visualization.

Figure S9. Representative bubble size distribution of foams in ethanol generated with 5wt% SiNP_F_{17}(1-3) and 1wt% IBu_7/F_{13}-POSS..

Figure S10. Optical images of ethanol foams with/without coverslip at different magnifications (the unit of scale bar is μm). Foaming conditions: 0.2-4wt% IBu_7/F_{13}-POSS, 4wt% SiNP_F_{17}(1-3), 1 g ethanol, 25 °C, stirring at 1500 rpm for 30 min, stabilization for 5 min before visualization.

Figure S11. Time-evolution of ethanol foams stabilized by the bi-particle system after stirring. a) Foam images with different times and IBu_7/F_{13}-POSS concentrations (5 min, 1h, 4h, 72h); b) Foam heights with different times and IBu_7/F_{13}-POSS concentrations; c) Normalized foam heights with different times and IBu_7/F_{13}-POSS concentrations. Foaming conditions: 0.2-2wt% IBu_7/F_{13}-POSS, 5wt% SiNP_F_{17}(1-3), 1 g ethanol, 25 °C, stirring at 1500 rpm for 30 min, stabilization for 5 min before visualization.

Figure S12. Optical images of ethanol foam stabilized by IBu_7/F_{13}-POSS + SiNP_F_{17}(1-3)_RB particles. Foaming conditions: 0.5wt% IBu_7/F_{13}-POSS, 8wt% SiNP_F_{17}(1-3)_RB, 1 g ethanol, 25 °C, stirring at 1500 rpm for 30 min, stabilization for 5 min before visualization.

Figure S13. Contact angle of SiNP_F_{17}(1-3), IBu_7/F_{13}-POSS and combined SiNP_F_{17}(1-3) + IBu_7/F_{13}-POSS (20:1 m/m) particle pellets with ethanol.

Figure S14. Dynamic surface tension tests of (a) ethanol and (b) benzyl alcohol at variable IBu_7/F_{13}-POSS concentration.

Figure S15. DLS tests of IBu_7/F_{13}-POSS and SiNP_F_{17}(1-3) particles (single and mixtures) dispersed in ethanol and THF. (a) 4wt% SiNP_F_{17}(1-3), 0.2wt% IBu_7/F_{13}-POSS, and particle mixtures with 4wt% SiNP_F_{17}(1-3) and variable IBu_7/F_{13}-POSS concentration in the range 0.2-2.0wt% in ethanol sonicated for 10 min before testing; (b) 4wt%SiNP_F_{17}(1-3) and 0.2wt% IBu_7/F_{13}-POSS particle mixture sonicated for 0 s, 3 s, 10 s, 1 min and 10 min in ethanol before DLS measurements. (c) 0.001wt%, 0.01wt%, and 0.1wt% IBu_7/F_{13}-POSS nanoparticles dispersed in THF sonicated for 10 min before testing.

Figure S16. Time-evolution of ethanol foamability by IBu_7/F_{13}-POSS + Pd@SiNP_F_{17}(1-3) particles during and after the aerobic oxidation reaction of BnOH. Reaction conditions: 1 g EtOH, 50 mg BnOH, 0.2 wt% IBu_7/F_{13}-POSS, 4 wt% Pd@SiNP_F_{17}(1-3), 60 °C, 1500 rpm, 1 h.

Figure S17. Foaming tests for benzyl alcohol and combinations of long chain aliphatic alcohols dissolved in ethanol using combined 1wt% IBu_7/F_{13}-POSS and 5wt%SiNP_F_{17}(1-3) particles. Foaming conditions: 5 wt% SiNP_F_{17}(1-3), 1% IBu_7/F_{13}-POSS, 1 g ethanol, 50 mg aliphatic alcohols (5 wt%), 25 °C, stabilization for 5 min before visualization, stirring at 1500 rpm for 30 min.

Figure S18. Foaming tests for (a) BnOH and (b) ethylene glycol. Foaming conditions: 4 wt% SiNP_F_{17}(1-3), 0.2% IBu_7/F_{13}-POSS, 1 g solvent, 25 °C, stabilization for 5 min before visualization, stirring at 1500 rpm for 30 min.

Figure S19. DLS tests of 0.2wt% IBu_7/F_{13}-POSS, 4wt% SiNP_F_{17}(1-3), and combined 0.2wt% IBu_7/F_{13}-POSS and 4wt% SiNP_F_{17}(1-3) particle mixtures in different solvents.

Figure S20. Schematic representation of particle recycling. (a) IBu_7/F_{13}-POSS and Pd@SiNP_F_{17}(1-3) are recycled together. (b) IBu_7/F_{13}-POSS and Pd@SiNP_F_{17}(1-3) are recycled separately.

Table S1. Average particle sizes measured by DLS for 0.2wt% IBu_7/F_{13}-POSS, 4wt% SiNP_F_{17}(1-3), and 0.2wt% IBu_7/F_{13}-POSS and 4wt% SiNP_F_{17}(1-3) particle dispersions in different solvents.

Solvent	0.2wt% IBu_7/F_{13}-POSS	0.2wt% IBu_7/F_{13}-POSS + 4wt% SiNP_F_{17}(1-3)	4wt% SiNP_F_{17}(1-3)
methanol	129	392	1180
isopropanol	147	499	1321
acetone	94	383	1072
dichloromethane	101	472	1265
octane	51	395	666
decane	75	404	592
dodecane	82	413	736
1-octene	122	481	957
Toluene	295	489	1491

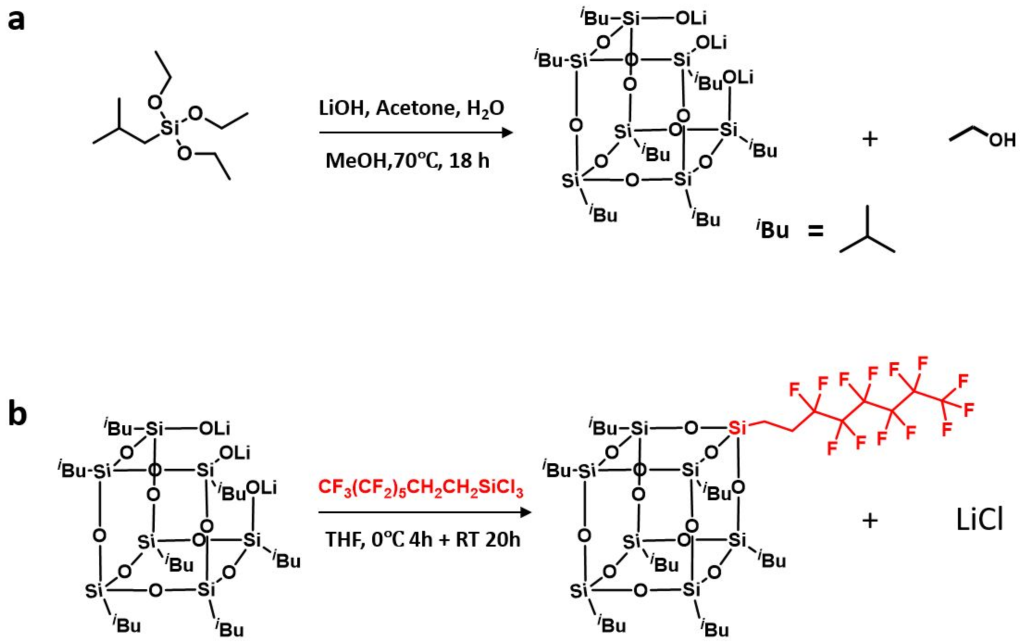

Figure S1. (a) Synthesis of IBu$_7$-POSS. **(b)** Synthesis of IBu$_7$/F$_{13}$-POSS.

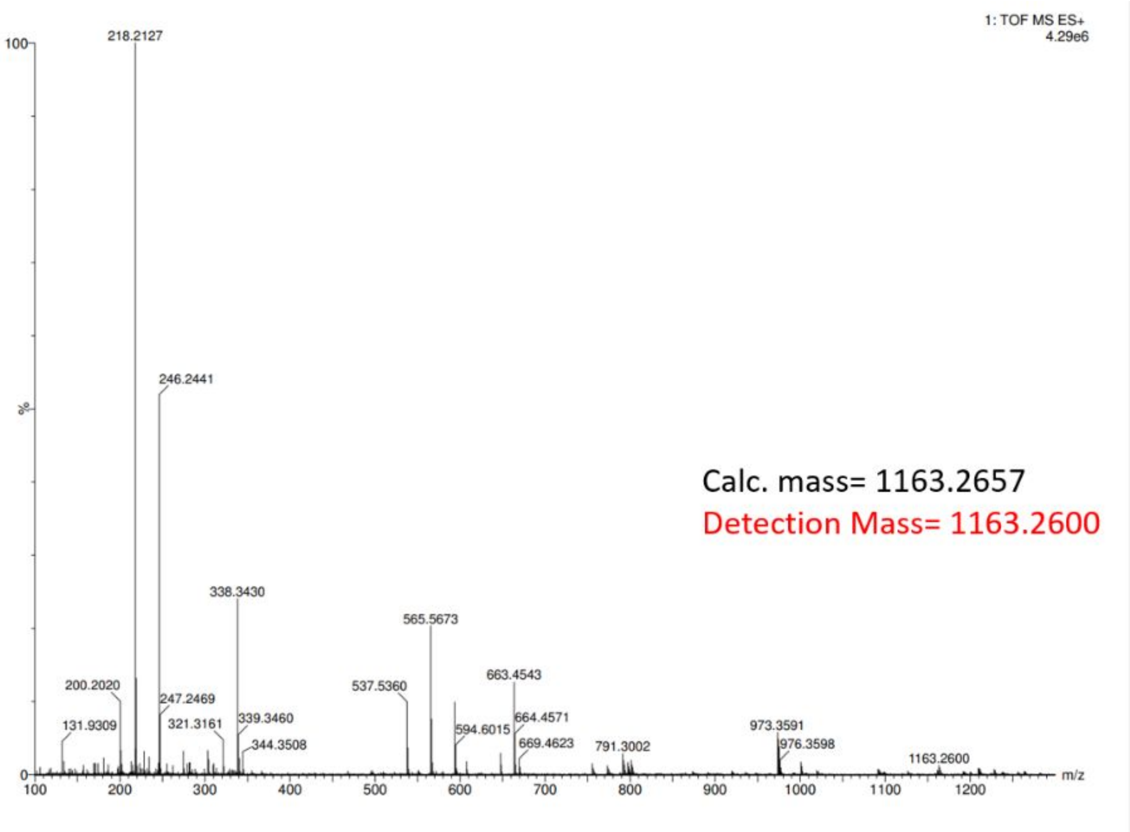

Figure S2. MS spectrogram of IBu_7/F_{13}-POSS.

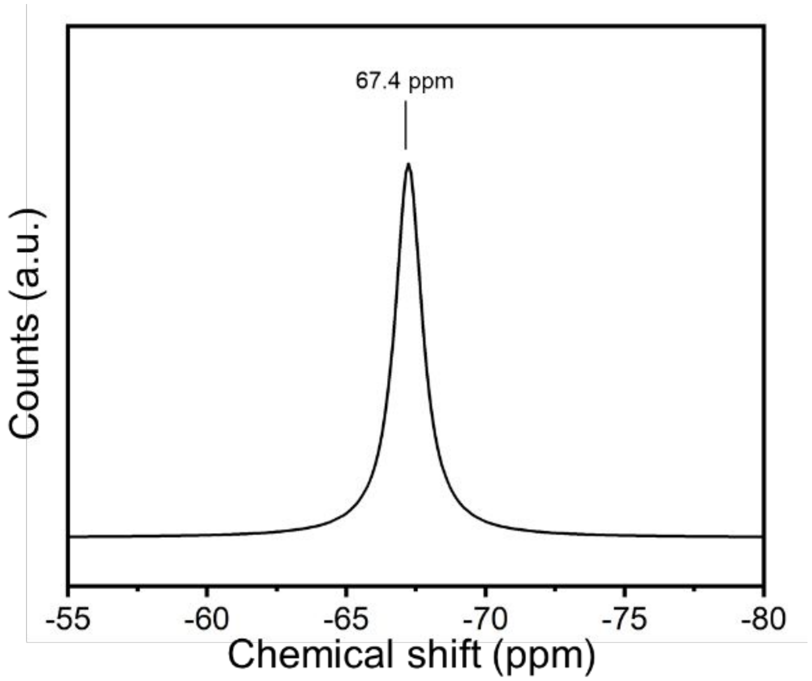

Figure S3. ^{29}Si NMR MAS spectrum of IBu_7/F_{13}-POSS.

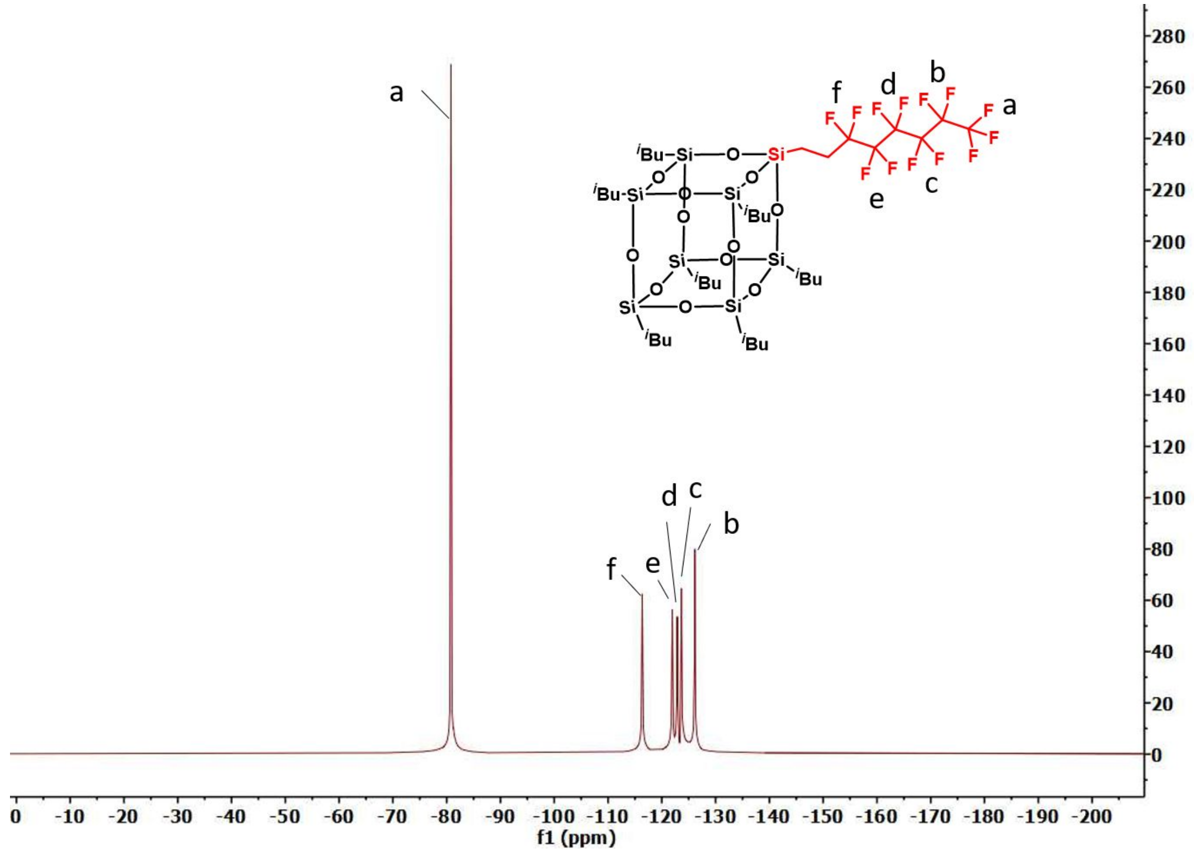

Figure S4. Liquid ^{19}F NMR spectrum of IBu_7/F_{13}-POSS.

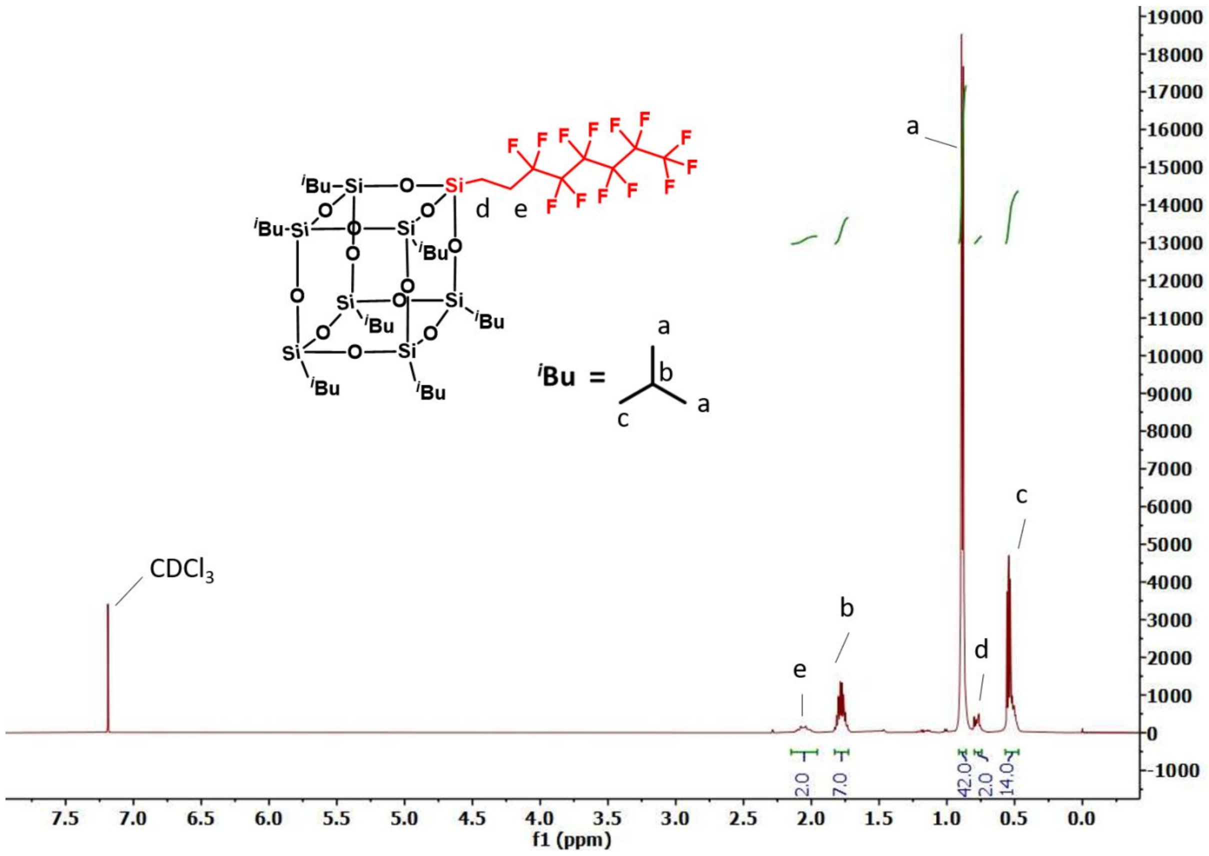

Figure S5. Liquid ^{1}H NMR spectrum of IBu_7/F_{13}-POSS. $CDCl_3$ was used as solvent.

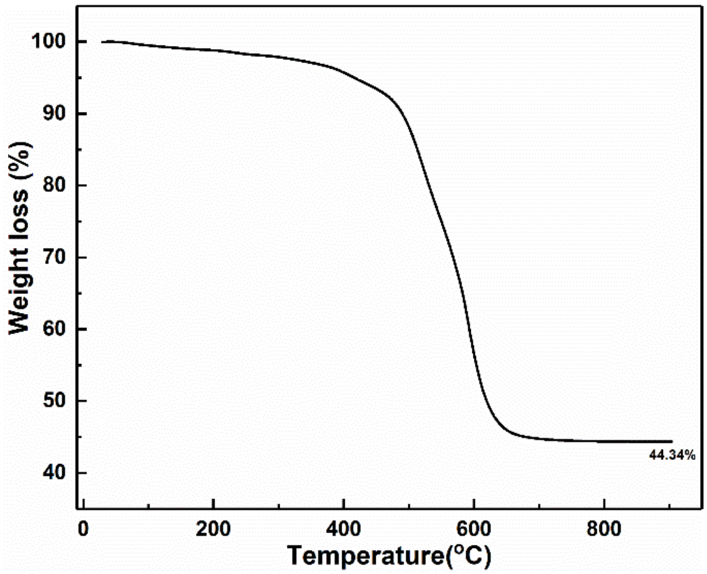

Figure S6. TGA profile of SiNP_F_{17}(1-3) particles

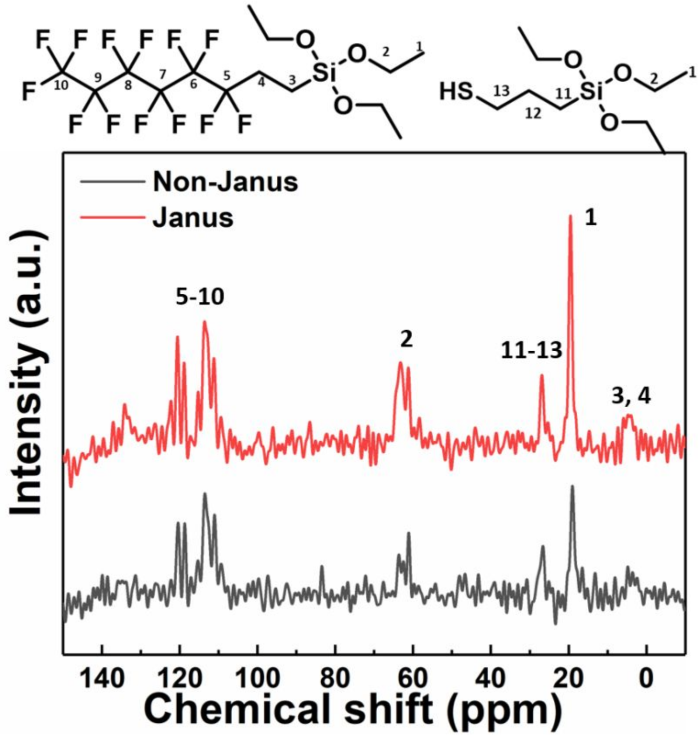

Figure S7. Solid-state ^{13}C NMR MAS spectra of JPs and Non-JPs.

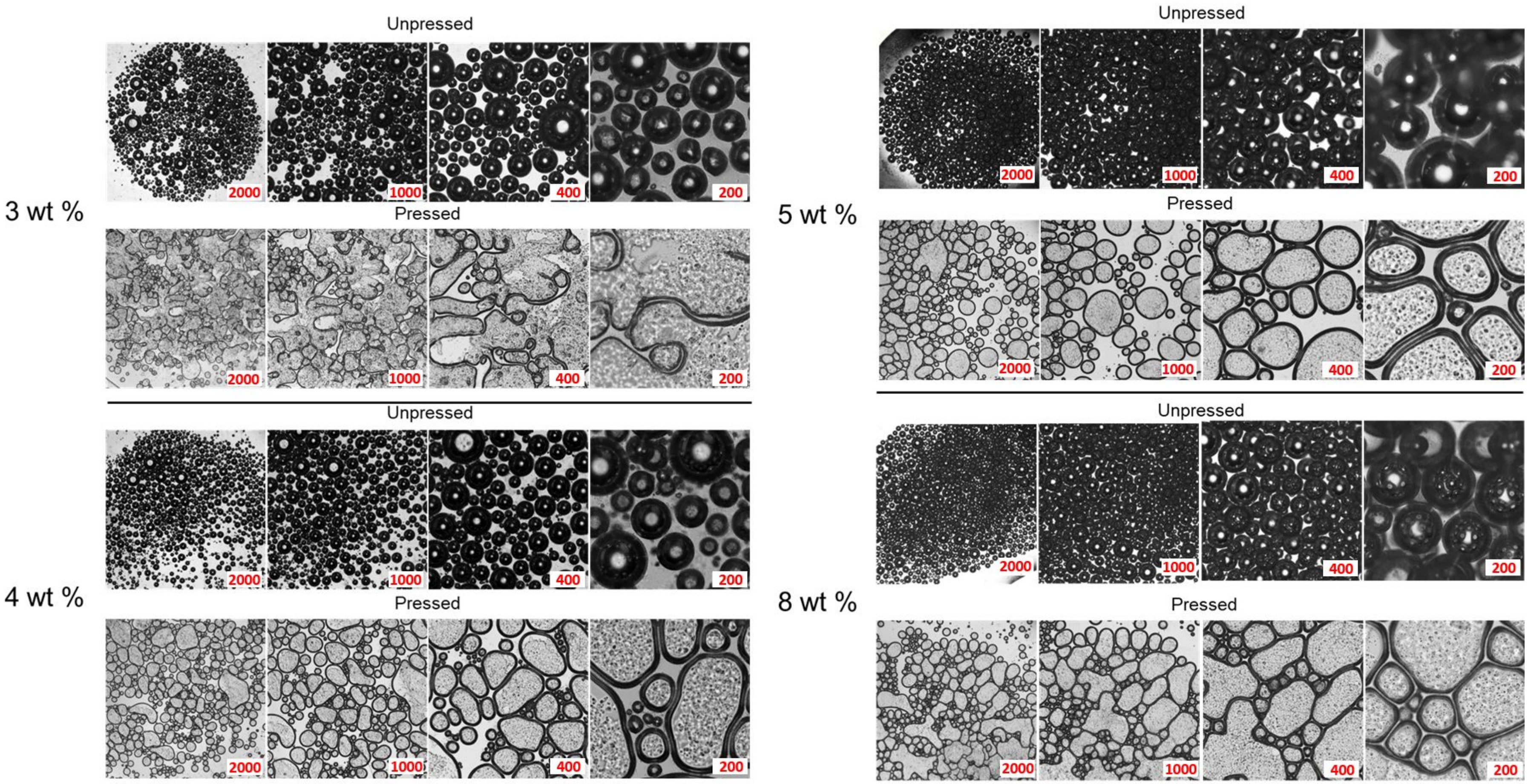

Figure S8. Optical images of ethanol foams with/without coverslip at different magnifications (the unit of scale bar is μm) after stirring. Foaming conditions: 1wt% IBu_7/F_{13}-POSS, 1-8wt% SiNP_F_{17}(1-3), 1 g ethanol, 25 °C, stirring at 1500 rpm for 30 min, stabilization for 5 min before visualization.

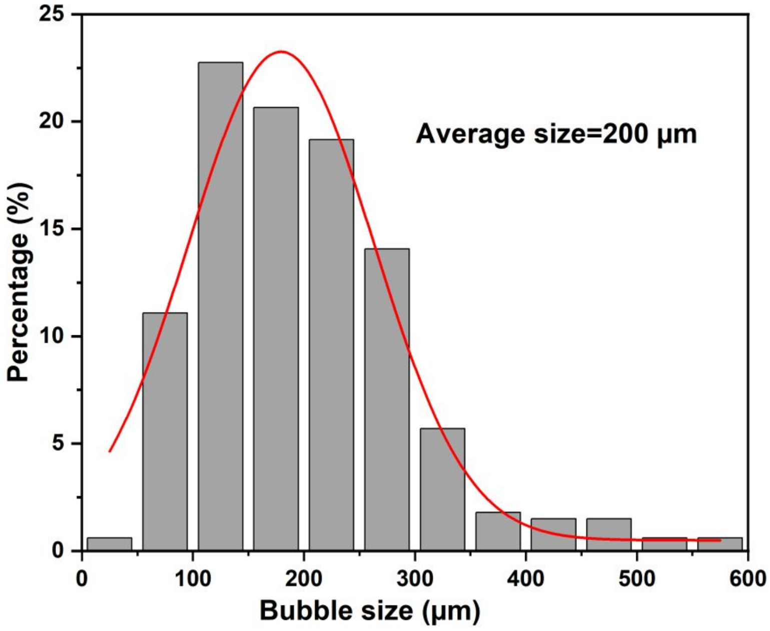

Figure S9. Representative bubble size distribution of foams in ethanol generated with 5wt% SiNP_F_{17}(1-3) and 1wt% IBu_7/F_{13}-POSS..

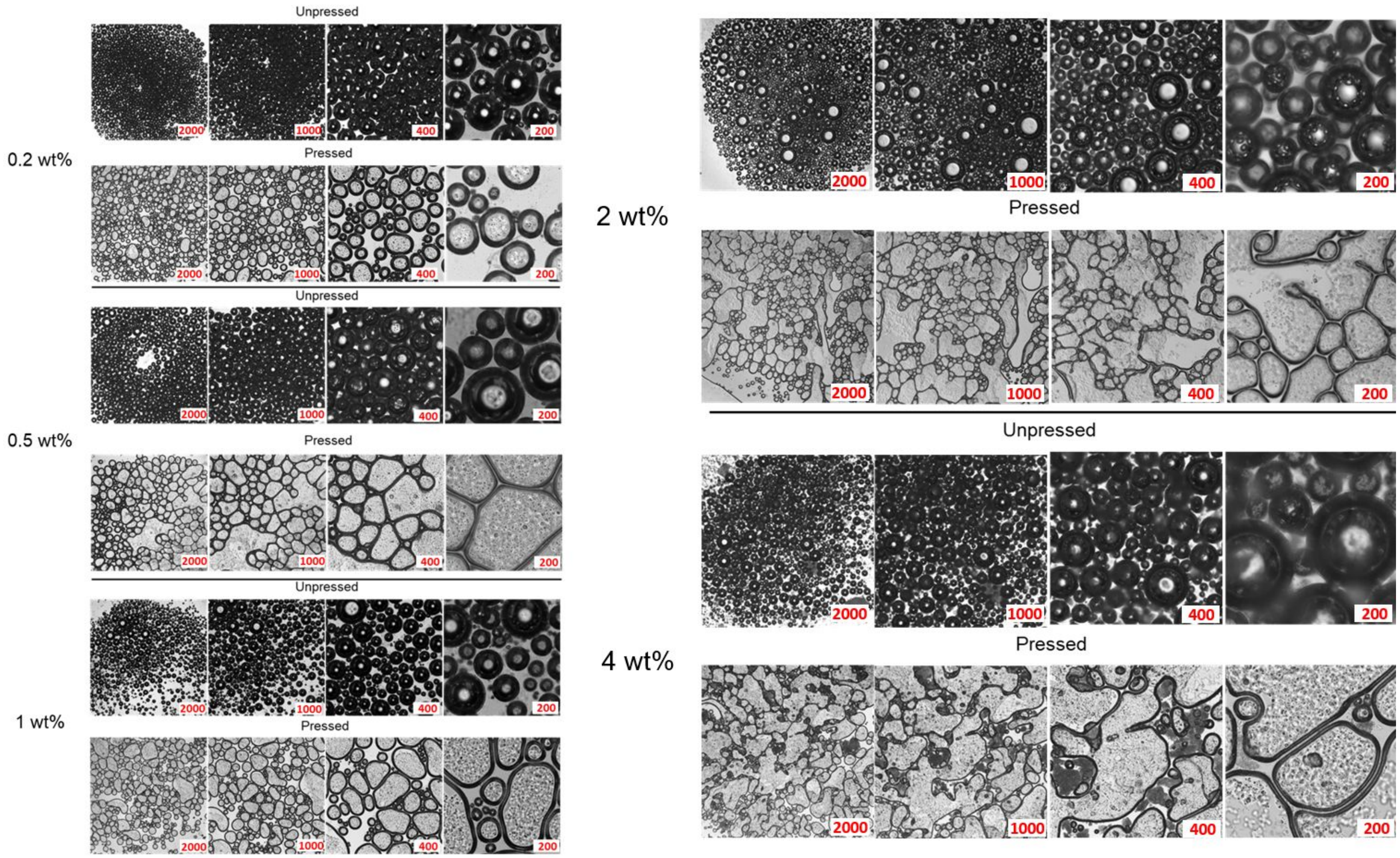

Figure S10. Optical images of ethanol foams with/without coverslip at different magnifications (the unit of scale bar is μm). Foaming conditions: 0.2-4wt% IBu_7/F_{13}-POSS, 4wt% SiNP_F_{17}(1-3), 1 g ethanol, 25 °C, stirring at 1500 rpm for 30 min, stabilization for 5 min before visualization.

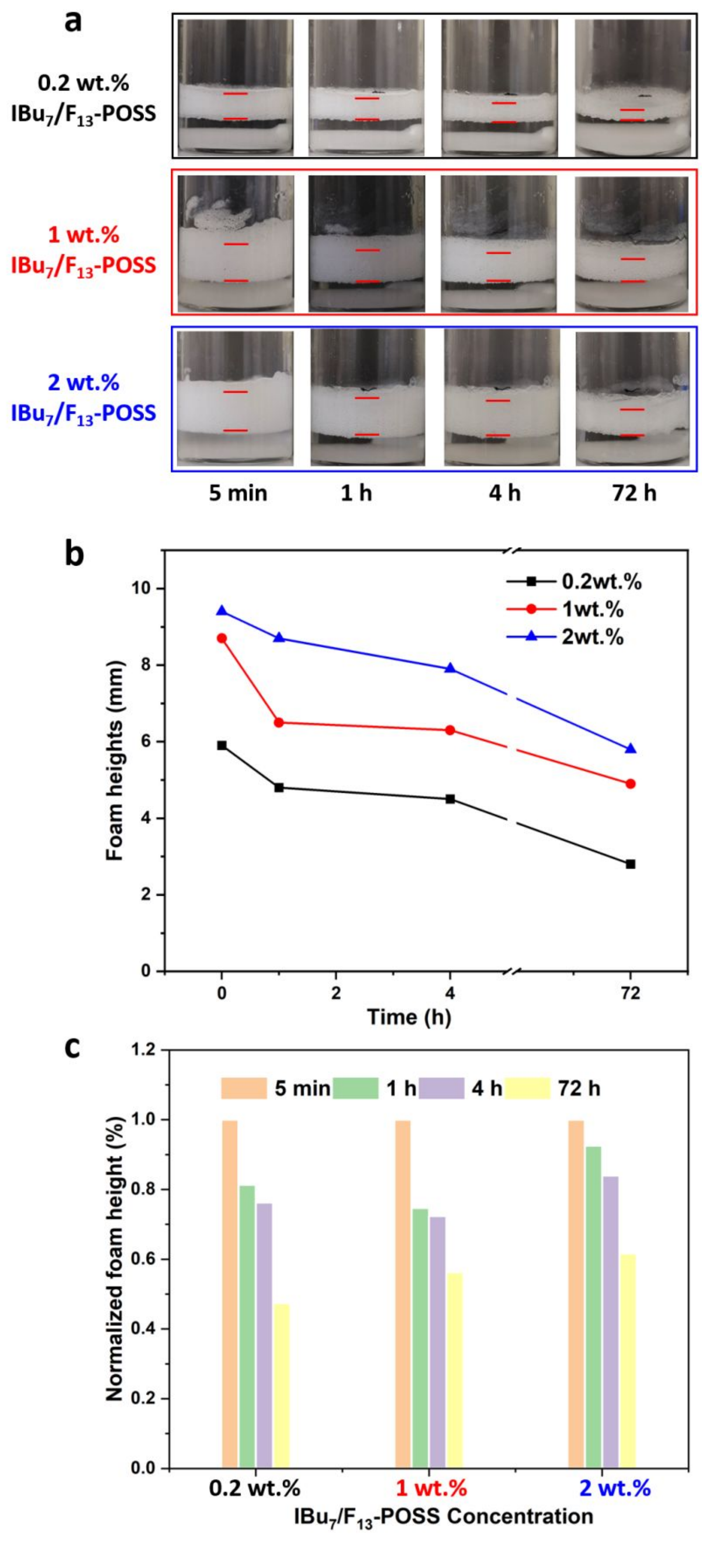

Figure S11. Time-evolution of ethanol foams stabilized by the bi-particle system after stirring. a) Foam images with different times and IBu_7/F_{13}-POSS concentrations (5 min, 1h, 4h, 72h); b) Foam heights with different times and IBu_7/F_{13}-POSS concentrations; c) Normalized foam heights with different times and IBu_7/F_{13}-POSS concentrations. Foaming conditions: 0.2-2wt% IBu_7/F_{13}-POSS, 5wt% SiNP_F_{17}(1-3), 1 g ethanol, 25 °C, stirring at 1500 rpm for 30 min, stabilization for 5 min before visualization.

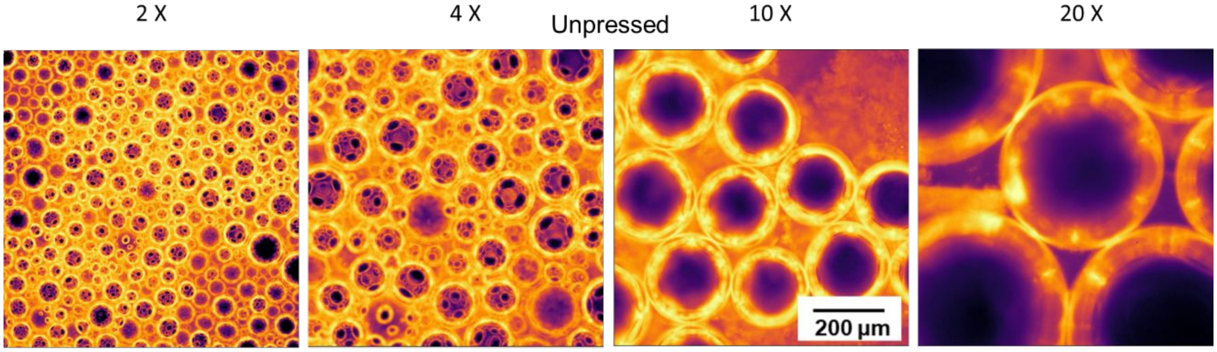

Figure S12. Optical images of ethanol foam stabilized by IBu_7/F_{13}-POSS + SiNP_F_{17}(1-3)_RB particles. Foaming conditions: 0.5wt% IBu_7/F_{13}-POSS, 8wt% SiNP_F_{17}(1-3)_RB, 1 g ethanol, 25 °C, stirring at 1500 rpm for 30 min, stabilization for 5 min before visualization.

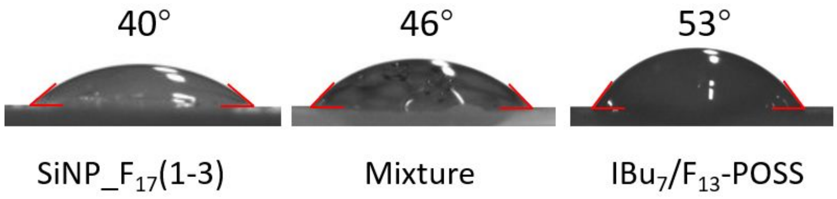

Figure S13. Contact angle of SiNP_F_{17}(1-3), IBu_7/F_{13}-POSS and combined SiNP_F_{17}(1-3) + IBu_7/F_{13}-POSS (20:1 m/m) particle pellets with ethanol.

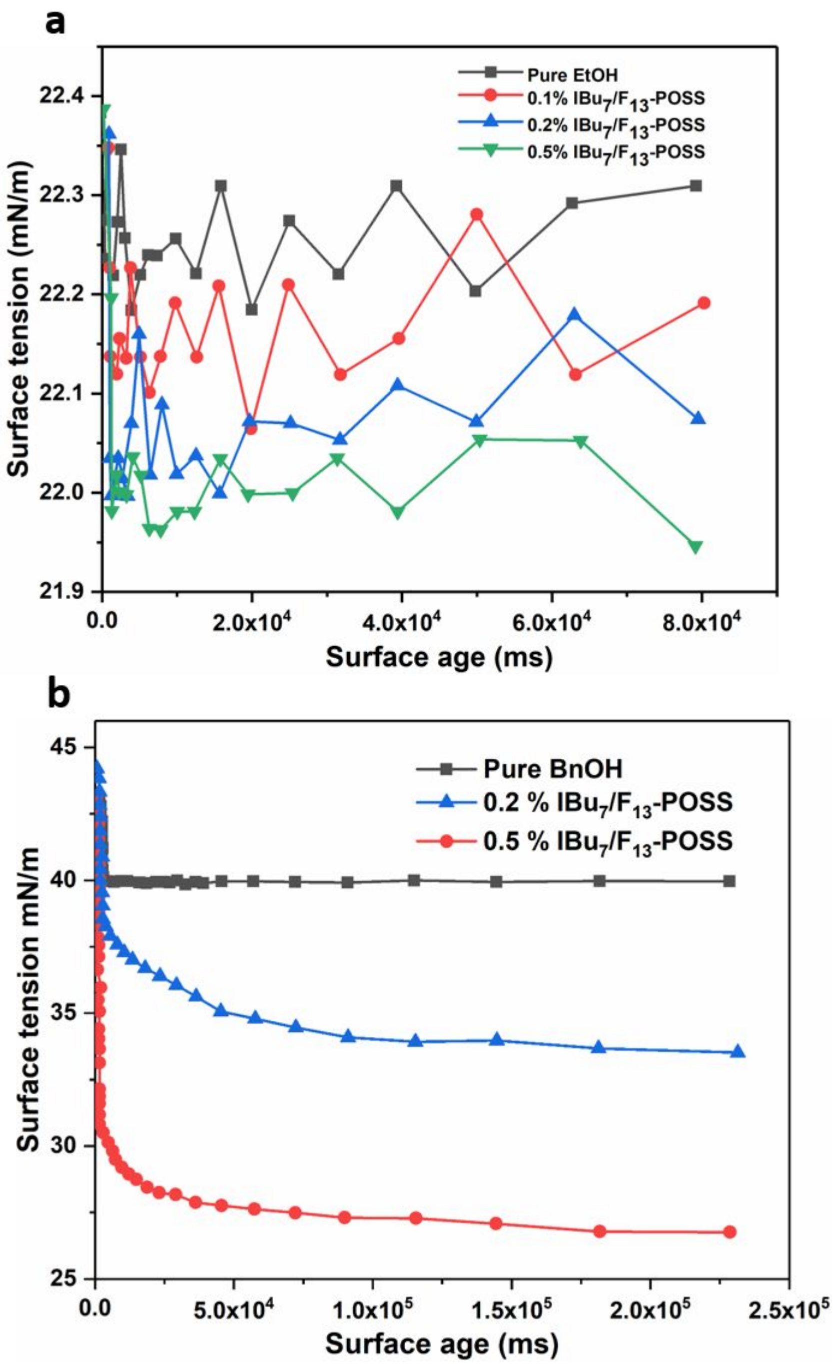

Figure S14. (a) Dynamic surface tension tests of (a) ethanol and (b) benzyl alcohol at variable IBu_7/F_{13}-POSS concentration.

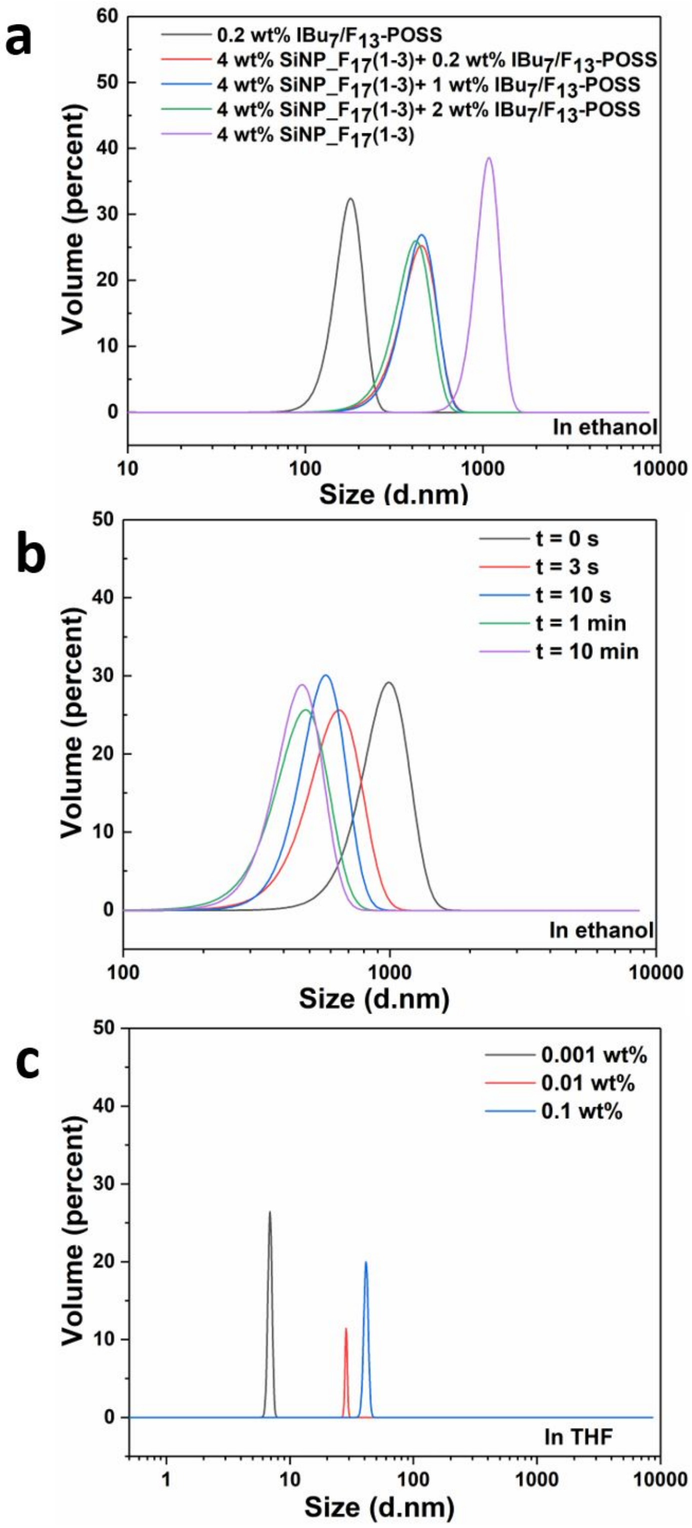

Figure S15. DLS tests of IBu_7/F_{13}-POSS and SiNP_F_{17}(1-3) particles (single and mixtures) dispersed in ethanol and THF. (a) 4wt% SiNP_F_{17}(1-3), 0.2wt% IBu_7/F_{13}-POSS, and particle mixtures with 4wt% SiNP_F_{17}(1-3) and variable IBu_7/F_{13}-POSS concentration in the range 0.2-2.0wt% in ethanol sonicated for 10 min before testing; (b) 4wt%SiNP_F_{17}(1-3) and 0.2wt% IBu_7/F_{13}-POSS particle mixture sonicated for 0 s, 3 s, 10 s, 1 min and 10 min in ethanol before DLS measurements. (c) 0.001wt%, 0.01wt%, and 0.1wt% IBu_7/F_{13}-POSS nanoparticles dispersed in THF sonicated for 10 min before testing.

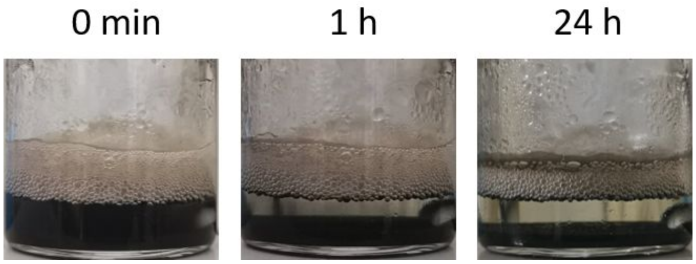

Figure S16. Time-evolution of ethanol foamability by IBu_7/F_{13}-POSS + Pd@SiNP_F_{17}(1-3) particles during and after the aerobic oxidation reaction of BnOH. Reaction conditions: 1 g EtOH, 50 mg BnOH, 0.2 wt% IBu_7/F_{13}-POSS, 4 wt% Pd@SiNP_F_{17}(1-3), 60 °C, 1500 rpm, 1 h.

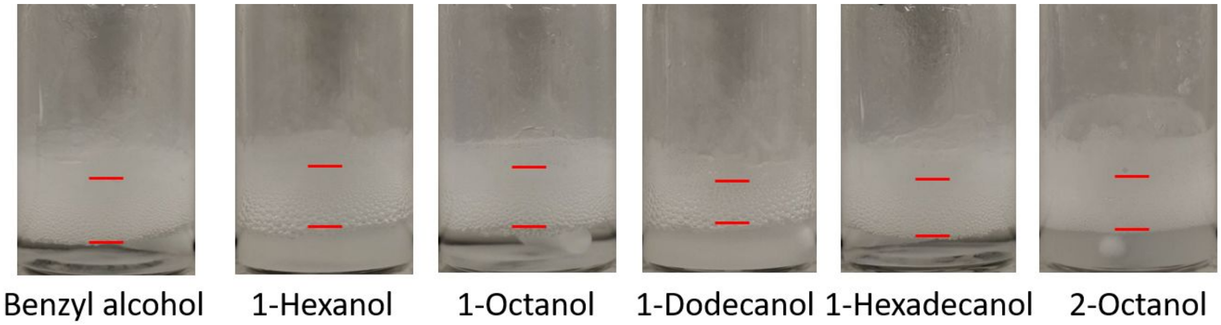

Figure S17. Foaming tests for benzyl alcohol and combinations of long chain aliphatic alcohols dissolved in ethanol using combined 1wt% IBu_7/F_{13}-POSS and 5wt%SiNP_F_{17}(1-3) particles. Foaming conditions: 5 wt% SiNP_F_{17}(1-3), 1% IBu_7/F_{13}-POSS, 1 g ethanol, 50 mg aliphatic alcohols (5 wt%), 25 °C, stabilization for 5 min before visualization, stirring at 1500 rpm for 30 min.

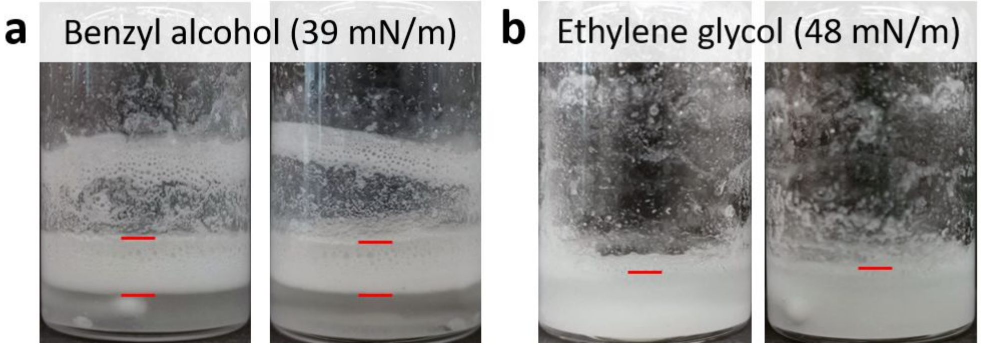

Figure S18. Foaming tests for (a) BnOH and (b) ethylene glycol. Foaming conditions: 4 wt% SiNP_F_{17}(1-3), 0.2% IBu_7/F_{13}-POSS, 1 g solvent, 25 °C, stabilization for 5 min before visualization, stirring at 1500 rpm for 30 min.

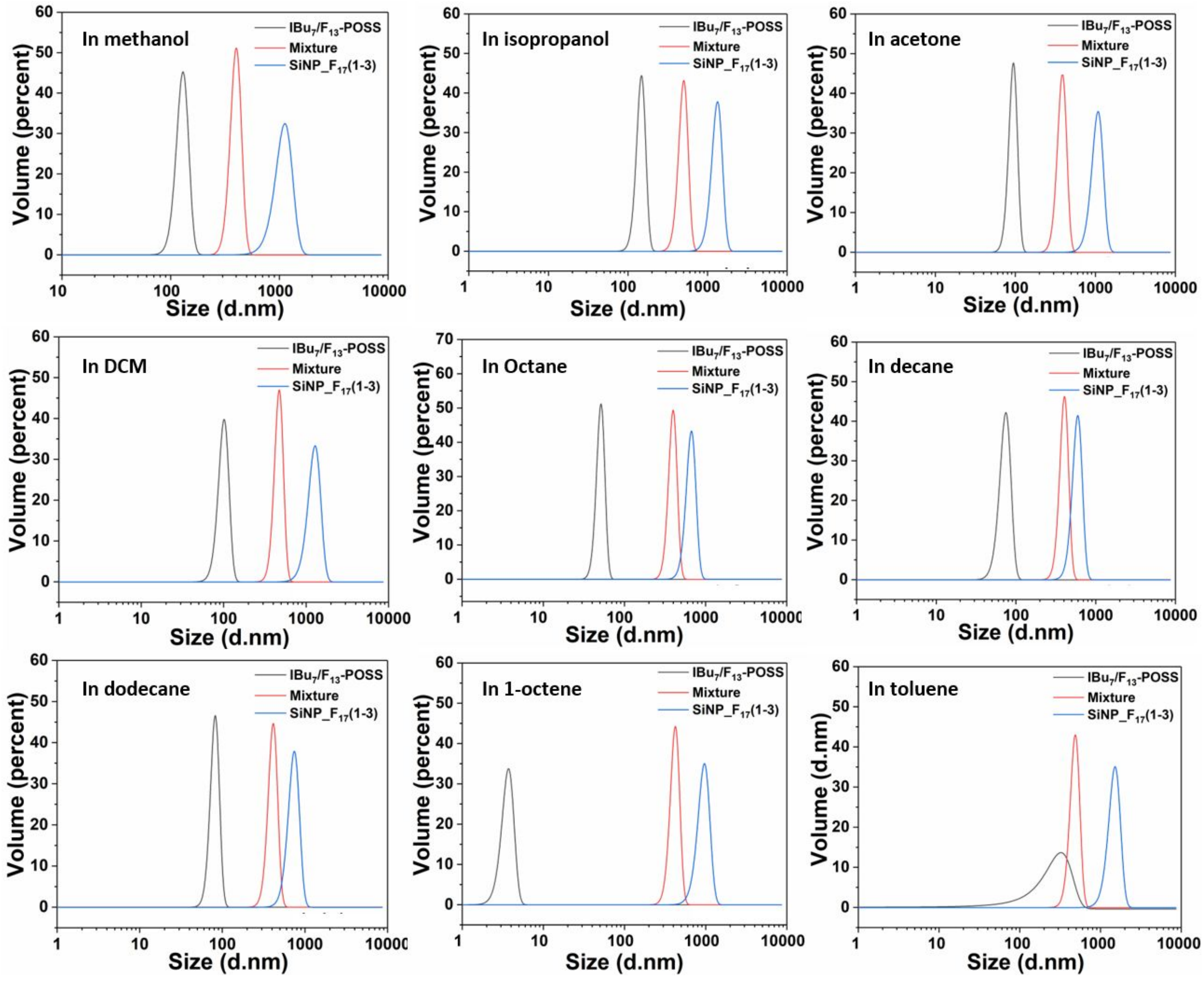

Figure S19. DLS tests using 0.2wt% IBu_7/F_{13}-POSS, 4wt% SiNP_F_{17}(1-3), and combined 0.2wt% IBu_7/F_{13}-POSS and 4wt% SiNP_F_{17}(1-3) particle mixtures dispersed in different solvents.

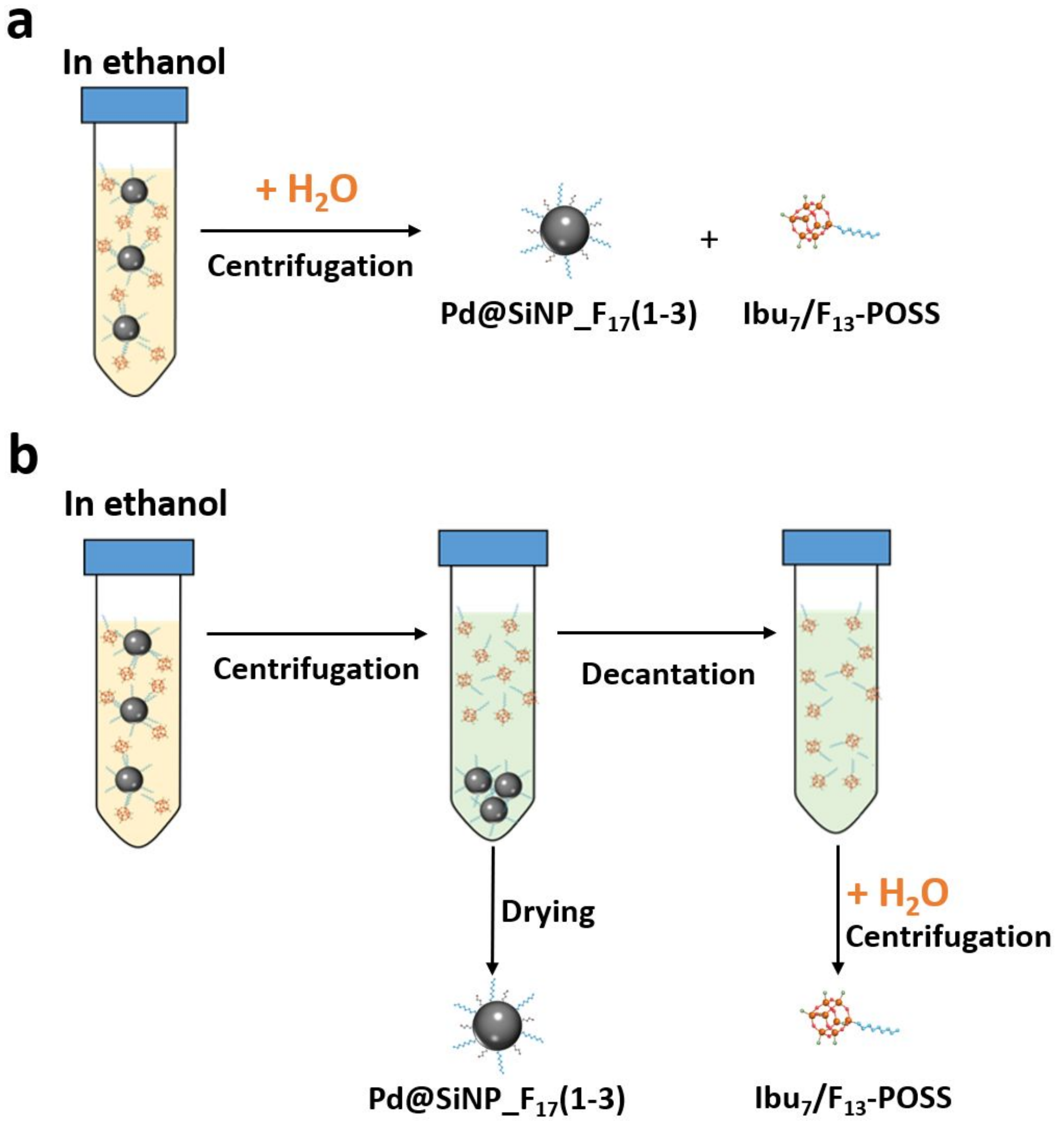

Figure S20. Schematic representation of particle recycling. (a) IBu$_7$/F_{13}-POSS and Pd@SiNP_F_{17}(1-3) are recycled together. (b) IBu$_7$/F_{13}-POSS and Pd@SiNP_F_{17}(1-3) are recycled separately.
